# Population allocation at the housing unit level: estimates around underground natural gas storage wells in PA, OH, NY, WV, MI, and CA

**DOI:** 10.1186/s12940-019-0497-z

**Published:** 2019-07-08

**Authors:** Drew R. Michanowicz, Samuel R. Williams, Jonathan J. Buonocore, Sebastian T. Rowland, Katherine E. Konschnik, Shaun A. Goho, Aaron S. Bernstein

**Affiliations:** 1 Center for Climate, Health and the Global Environment, Harvard T.H. Chan School of Public Health, 401 Park Drive, Landmark Center 4th floor west suite 415E, Boston, MA 02215 USA; 2 Department of Environmental Health, Boston University, Boston, MA 02215 USA; 3 Department of Environmental Health Sciences, Columbia University, New York City, NY 10027 USA; 4 Nicholas Institute for Environmental Solutions, Duke University, Durham, NC 27708 USA; 5 Emmett Environmental Law & Policy Clinic, Harvard Law School, Cambridge, MA 02138 USA; 6 Division of General Medicine, Boston Children’s Hospital, Boston, MA 02115 USA

**Keywords:** Population at risk, Oil and gas, Underground natural gas storage, Safety, Environmental health, U.S. Census, Setbacks

## Abstract

**Background:**

Spatially accurate population data are critical for determining health impacts from many known risk factors. However, the utility of the increasing spatial resolution of disease mapping and environmental exposures is limited by the lack of receptor population data at similar sub-census block spatial scales.

**Methods:**

Here we apply an innovative method (Population Allocation by Occupied Domicile Estimation – ABODE) to disaggregate U.S. Census populations by allocating an average person per household to geospatially-identified residential housing units (RHU). We considered two possible sources of RHU location data: address point locations and building footprint centroids. We compared the performance of ABODE with the common proportional population allocation (PPA) method for estimating the nighttime residential populations within 200 m radii and setback areas (100 – 300 ft) around active underground natural gas storage (UGS) wells (*n* = 9834) in six U.S. states.

**Results:**

Address location data generally outperformed building footprint data in predicting total counts of census residential housing units, with correlations ranging from 0.67 to 0.81 at the census block level. Using residentially-sited addresses only, ABODE estimated upwards of 20,000 physical households with between 48,126 and 53,250 people living within 200 m of active UGS wells – likely encompassing the size of a proposed UGS Wellhead Safety Zone. Across the 9834 active wells assessed, ABODE estimated between 5074 and 10,198 more people living in these areas compare to PPA, and the difference was significant at the individual well level (*p* = < 0.0001). By either population estimation method, OH exhibits a substantial degree of hyperlocal land use conflict between populations and UGS wells – more so than other states assessed. In some rare cases, population estimates differed by more than 100 people for the small 200 m^2^ well-areas. ABODE’s explicit accounting of physical households confirmed over 50% of PPA predictions as false positives indicated by non-zero predictions in areas absent physical RHUs.

**Conclusions:**

Compared to PPA – in allocating identical population data at sub-census block spatial scales –ABODE provides a more precise population at risk (PAR) estimate with higher confidence estimates of populations at greatest risk. 65% of UGS wells occupy residential urban and suburban areas indicating the unique land use conflicts presented by UGS systems that likely continue to experience population encroachment. Overall, ABODE confirms tens of thousands of homes and residents are likely located within the proposed UGS Wellhead Safety Zone – and in some cases within state’s oil and gas well surface setback distances – of active UGS wells.

**Electronic supplementary material:**

The online version of this article (10.1186/s12940-019-0497-z) contains supplementary material, which is available to authorized users.

## Background

Estimates of the spatial distribution of human populations supports planning and decision-making across a range of domains, including public health, transportation, sustainable development, and climate change resilience [1–3]. Public health research often relies on estimates of the population at risk (PAR), or the number of individuals who may experience known risk factors of disease [4–8], as well as natural and manmade hazards [2, 9–11]. Such estimates support epidemiology, exposure assessment, and first-order risk analysis research that assesses the potential impact of an exposure. However, coarse spatial resolution of population data, relative to exposure and hazard location data, has limited advances in estimating PAR for a variety of hazards.

In the United States, population data are publicly available at aggregated aerial units (e.g., census blocks and tracts), and typically represent nighttime residential population since census surveys capture where respondents reside, rather than time spent working and traveling. Unfortunately, when this population data is applied to health research, these data lead to the classic aerial unit problem (AUP), referring to the statistical biasing observed in a spatial analysis when the geographic context or scale is modified [12]. Studies that rely on census data or similar data to estimate PAR are constrained by the AUP and therefore are required to make assumptions about the spatial structure of populations – typically the assumption that populations are homogeneously distributed within aerial units (i.e., proportional weighting). Such assumptions are often not explicitly stated, are rarely valid, introduce uncertainly, and are increasingly important for spatially discrete phenomena and risk regimes such sea level rise, hazardous containment areas, audible warning systems, and explosion blast radii, among others.

Dasymetric mapping techniques can improve proportional population allocation (PPA) methods by allocating census population counts to likely habitable land uses [13–15], but still estimates populations at an area-scale, and therefore relies upon the assumption of uniform population density within those habitable areas. Population allocation at a discrete location such as a residential housing unit (defined by the U.S. Census as a house, apartment, group of rooms, or a single room occupied or intended for occupancy as a separate living quarters) can attenuate the AUP by avoiding to work at area-scales and thus avoids the assumption of uniform population density across space. Remote sensing technologies can provide more accurate representations of population spatial heterogeneity [2, 16–19], and have been utilized to estimate populations at the individual building/household scale [20–23]. However, building/household-level predictions have been limited to small study areas, and widescale population allocation to discrete locations such as physical buildings been ultimately limited by the availability and quality of residential housing location data.

Here, we first test the efficacy of three geospatial data sources to proxy for residential housing unit (RHU) locations: building footprint data, and two publicly-available address datasets (OpenAddresses.io and the U.S. Department of Transportation’s National Address Database). We then present the population allocation by occupied domicile estimation (ABODE) method that disaggregates census block data to discrete RHUs and compare it to the common proportional population allocation (PPA) method by enumerating populations living within 200 m of active underground natural gas storage wells (UGS) in six U.S. States. Additionally, we apply ABODE to estimate the number of households and populations within very small areas around UGS wells – state oil and gas surface setback area regulations where applicable. While some studies have provided theoretical evidence that such setbacks – the minimum distance which an occupied structure must be set back from a well – may not be protective of health or safety [24, 25], few have relied on geospatial physical dwelling data to describe extant land use conflicts that could result from population encroachment and the general lack of reverse setback rules.

PAR estimates around UGS wells provide a good test case for ABODE for several reasons. First, UGS wells are geographically dispersed across 32 U.S. states. Recent obsolescence issues identified at UGS facilities (e.g., single-point-of-failure well designs) have placed new scrutiny on the hazards they may pose to nearby populations [26–28]. Second, the Interagency Task Force on Natural Gas Storage Safety, which was formed after the 2015 Aliso Canyon leak, recommended that “stakeholders should collect and analyze data on the proximity of UGS facilities to population centers to help better quantify some of the risk factors” [27]. Our development of spatially-precise PAR estimates directly responds to this request. Third, though many UGS facilities were originally sited at city limits decades prior, population encroachment from urban and suburban sprawl has likely created an understudied area of potential land use conflicts and PAR to low probability/high impact loss of containment events. Fourth, although numerous studies have attempted to quantify populations potentially at increased risk of adverse health effects due to their proximity to active oil and gas operations [11, 29], UGS wells have largely been excluded from these analyses due to poor well location data availability. Finally, small area population estimates are also informative for assessing consistency with a Wellhead Safety Zone that the Oak Ridge National Laboratory has recently proposed for UGS wells based upon individual well diameter and operating pressure [30].

## Methods

### Study areas

First, we selected UGS wells in states based upon: 1) prevalence of UGS operations (e.g., total facilities, wells, and working gas); 2) total populations of census blocks in which UGS wells are located; 3) address and building data availability and quality; 4) a history of UGS incidents; 5) facility- and well ages; and; 6) variability in legislative building setback distances. Based on our criteria, we chose UGS wells in Pennsylvania (PA), Ohio (OH), New York (NY), West Virginia (WV), Michigan (MI), and California (CA). For each of these states, we compared PAR estimates from ABODE and PPA under two search distances. The first search distance was 200 m, which corresponds to the U.S. Federal Pipeline Hazardous Materials Safety Administration (PHMSA) high consequence area designations intended to mitigate harms from pipeline explosions to adjacent communities [49 U.S.C. § 192.5(a) [1]]. PAR estimates for 200 m radii also likely represent a lower bound of a proposed Wellhead Safety Zone for active UGS wells- based on the area that would on average exceed a 5000 Btu/hr-ft^2^ radiant heat flux during the first 30 s of an explosion [30]. The second search distance varied by state, and represented each state’s individual surface setback distance, which ranged from 100 to 300 ft. NY (100 ft), OH (100 ft), PA (200 ft), MI (300 ft), and WV (200 ft) (CA does not have building setback rules) (See Additional file 1: Table S4 for additional detail). Surface setbacks are one regulation-based strategy that is intended to protect the health and safety of residents by setting an exclusion area around a well that shall not contain an occupied dwelling. While these setback rules are typically intended for new producing wells, applicability to certain well types including UGS wells varies by state.

### UGS well data

We used previously published data on UGS facilities and wells from Michanowicz [26]. Well counts included all active wells connected to active UGS facilities from the April 2016 Energy Information Administration-191 M *Monthly Underground Gas Storage Report* [31]. The six states selected contain 179 UGS facilities and 9834 active wells, representing 47% of facilities and 61% of active UGS wells nationwide. The UGS facilities in these states handle on average 2.1 Bcf, totaling 44% of national stored gas capacity.

### Spatial accuracy of UGS well location data by visual inspection

To assess the spatial accuracy of the publicly available well data, we performed a visual verification of wellhead locations for a simple random lottery selection of 5% of wells in each state. Reported well locations were compared to visual appearance in aerial images across seasons from two sources: the 2.5 - 15 m TerraColor imagery (Earthstar Geographics LLC, imagery) from the Landsat 8 satellite (2013–2017), and the 0.5–1.0 m imagery from the National Agriculture Imagery Program (NAIP) administered every 3 years by the U.S. Department of Agriculture Farm Services Agency. For each well, we either calculated an offset distance between the reported coordinates and the observed location or reported the well as “unidentified.” We took measures to prevent misclassifying well location accuracy during the visual inspection. If a well could not be identified within a search area of 1000 ft^2^ from the administrative coordinates or could not be distinguished among multiple wells in the area, then the location was reported as “unidentified.” The percentage of wells visually verified, and their median offset distances are presented in Table 1.

### Population allocation by occupied domicile estimation (ABODE)

ABODE relies on residential housing unit locations to provide “anchor points” onto which average persons per household from census block estimates can be disaggregated. This process first required quality assessments of candidate address and building location data. Not all addresses and buildings represent residential dwellings, so a land use model first was applied to remove candidate address and building points that resided in commercial, industrial, or institutional parcels (See Additional file 1: Table S2 for complete listing of land use types). The remaining candidate locations were then assigned as ‘residential housing units’ (RHUs) to represent discrete locations of nighttime residential populations. Because ABODE relies on identification of physical dwellings, both populations and counts of occupied dwellings in an area can be reported.

### Address and building location data

Residential housing units are defined by the U.S. Census as a house, apartment, group of rooms, or a single room occupied or intended for occupancy as a separate living quarters. Candidate data consisted of two types – address coordinate points, and geospatial building footprints/centroids. Address location coordinate points were available (as of October 23, 2017) from the US Department of Transportation’s National Address Database (NAD) (NY, OH) and OpenAddressess.io (PA, WV, MI) originally sourced from state geographic information systems departments, the U.S. Postal Service, and county property parcel datasets [32, 33]. Geospatial building footprints and centroids were available via academic use waiver for various parts of the country from BuildingFootprintUSA (BFUSA, Albany, NY). Address location data were not available for California and certain counties of PA and MI (e.g., twelve PA counties missing address data), and therefore only building footprint data or manual entry could be applied in these well areas. Notably, address data from the NAD and OpenAddresses.io were assumed to account for multi-residential unit buildings (i.e., multi-family apartment complexes) through duplicate address coordinate points, whereas building location data only provided the location of a building, without indication of whether it was a single residence or a multi-unit building.

### Residential housing units

In order to censor only potential RHU’s within residential areas, we applied a dasymetric filter. Based on the 2010 land use parcel model from Theobald [34] at 30 × 30 m resolution, candidate address and buildings that intersected with built-up (BU) commercial, industrial and institutional use grid cells were excluded from the final RHU list (see Additional file 1: Table S2). Additional file 1: Table S2 also shows the counts of land use parcels that correspond with all addresses located within 200 m of an active UGS well. RHU counts from each method were summed at the census block level and compared to RHU counts from the 2010 U.S. Census. This comparison was performed for all census blocks within two randomly selected counties of each NY, OH, and PA that contained complete coverage for both address and building data. In predicting total counts of housing units from the census at the census block level, we presented one-to-one plots, correlation coefficients (Spearman), mean absolute percentage errors (MAPE – with zero predictions removed) and FAC2 which describes the fraction of data that satisfy

1 $$ 0.5\le \frac{Candidate\ RHUs}{Census\ housing\ units}\le 2.0 $$

The dataset that best predicted U.S. Census RHU counts at the census block level across the two counties of each NY, OH, and PA was selected for the subsequent ABODE estimations for all six states.

### Spatial accuracy of residential housing unit locations at state setback distances

To assess the accuracy of the building and address location data, and resolve reliable RHU estimates within state setback distances, a visual inspection was performed for only the well-areas that initially overlapped with a candidate RHU corresponding to applicable state surface setback rules. UGS wells that did not initially intersect with candidate RHU data were not visually inspected. Therefore, RHUs were visually verified around UGS wells in NY (100 ft), OH (100 ft), PA (200 ft), MI (300 ft), and WV (200 ft). Though CA does not have building setback rules, all CA RHUs were manually imputed due to limited address/building data. Visual inspection utilized similar aerial imagery products described above and entailed removing RHUs that did not appear to represent an inhabitable structure, and no RHUs were added during this inspection process. Visually-verified RHUs were then carried through to subsequent ABODE 200 m RHU counts and population estimates. Generally, address location data correspond to one of four distinct physical location types: 1) physical buildings (e.g., building footprint centroids), 2) land parcels containing a building, 3) land parcels without building(s), and 4) point at nearest street segment (e.g., mail box location). Generally, address points from criteria “3” were the only candidate RHUs removed. See Additional file 1: Figure S5 for examples of address location types described herein, and Additional file 1: Table S4 for results of visual verification. Address and residential housing unit data assumptions and anticipated biases are presented in Additional file 1: Table S1.

### Population at risk

For each well, we first defined an area (*j*)*,* representing a radial buffer area around each well drawn using the 200 m search radii. For each given area *j*, we first identified the number of residential housing units within each census block *i*, partially or completely contained within the area *j,* as denoted by *RHU*_*ij*_ in eq. (2).

2 $$ {\sum}_{i=1}^n{RHU}_{ij} $$

The U.S. Census provides the average ‘person per household’ (i.e., “average household size”- T064_001 from the U.S. Census of Population and Housing Summary File) at census block *i* denoted by ${\overline{pph}}_i$ which equates to the total population of census block *i* (excluding populations in group quarters) divided by the total number of housing units within that same block. Total population of an area *j* can then be calculated by summing the product of the count of residential housing units that intersect with area *j* multiplied by the average person per household for census block *i* at as show in eq. (3).

3 $$ {Pop}_j={\sum}_{i=1}^n{RHU}_{ij}\ast {\overline{pph}}_i $$

It is important to note that eq. (3) fails to account for populations residing in group quarters (e.g., prisons, university dorms). Certain care should be taken at the outset to account for the presence of group quarters populations in *j* areas of interest that intersect with census group quarters populations. Within each census block, the number of RHUs that contributed to the PAR was capped at the number of housing units reported by the census to help avoid possible inflation from including non-residential addresses or post 2010 housing unit growth – referred to “capped RHU/ABODE” henceforth. Uncapped population estimates were also reported for comparison (“ABODE”) and may reflect new population- and residential housing unit growth between 2010 and 2018. The publishing dates of address data typically at county-level ranged between 2009 to 2018 (See Additional file 1: Table S5).

ABODE estimates were then compared to a common proportional population allocation (PPA) area weighting approach that allocates census population density (i.e., persons per square mile) to an area of interest. To calculate the number of people in area *j* using PPA, first all 200 m well-area polygons were dissolved to prevent doubling counting then intersected with census block polygons. The overlapping census block segments were then clipped to the well-areas and new geometric areas were calculated. These calculated areas were then multiplied by the population density of the residing census block to resolve a population estimate that could be summed across all clipped census block segments. To compare PPA and ABODE estimates, we presented well-level prediction differences (ABODE minus PPA) across states with a focus on disagreement scenarios that highlights PPA false positives (i.e., PPA predicts positive populations, yet no RHUs are present) (Fig. 2). We also reported one-to-one plots (Additional file 1: Figure S3), tests of equal variances, FAC2, and fractional bias (FB) explained by:

4 $$ \mathrm{FB}=\frac{1}{n}\left(\frac{\sum_1^n ABODE- PPA}{\sum_1^n\frac{ABODE+ PPA}{2}}\right) $$

where ABODE and PPA represent populations predictions for individual well-areas for *n* wells per state. Statistics were performed using STATA v. 14.2 (College Station, TX), and R v. 3.2.2. (R Core Team, 2019).

## Results

The accuracy of publicly available UGS well location data varied by state. MI and CA well locations were more accurate than northeastern states as indicated by proportions of unidentified wells (beyond a 1000 ft. search radius) and shorter offset distances in MI and CA from visual inspection via aerial imagery (Table 1).

**Table 1:** Visual verification of well locations

State	Active UGS facilities // wells	Wells visually verified^a^	Visually unidentified wells^a^	Median (SD) visual offset distance (ft.)
CA	12 // 346	17	5	10.9 (7.4)
MI	39 // 2394	119	4	10.9 (8.2)
NY	26 // 972	48	4	52.3 (174.8)
OH	21 // 3318	166	26	55.5 (117.3)
PA	46 // 1332	67	11	62.7 (126.5)
WV	25 // 1472	73	30	82.8 (131.3)

^a^Verified and unidentified wells combined equal 5% random sample of active UGS wells per state

**Fig. 1:**
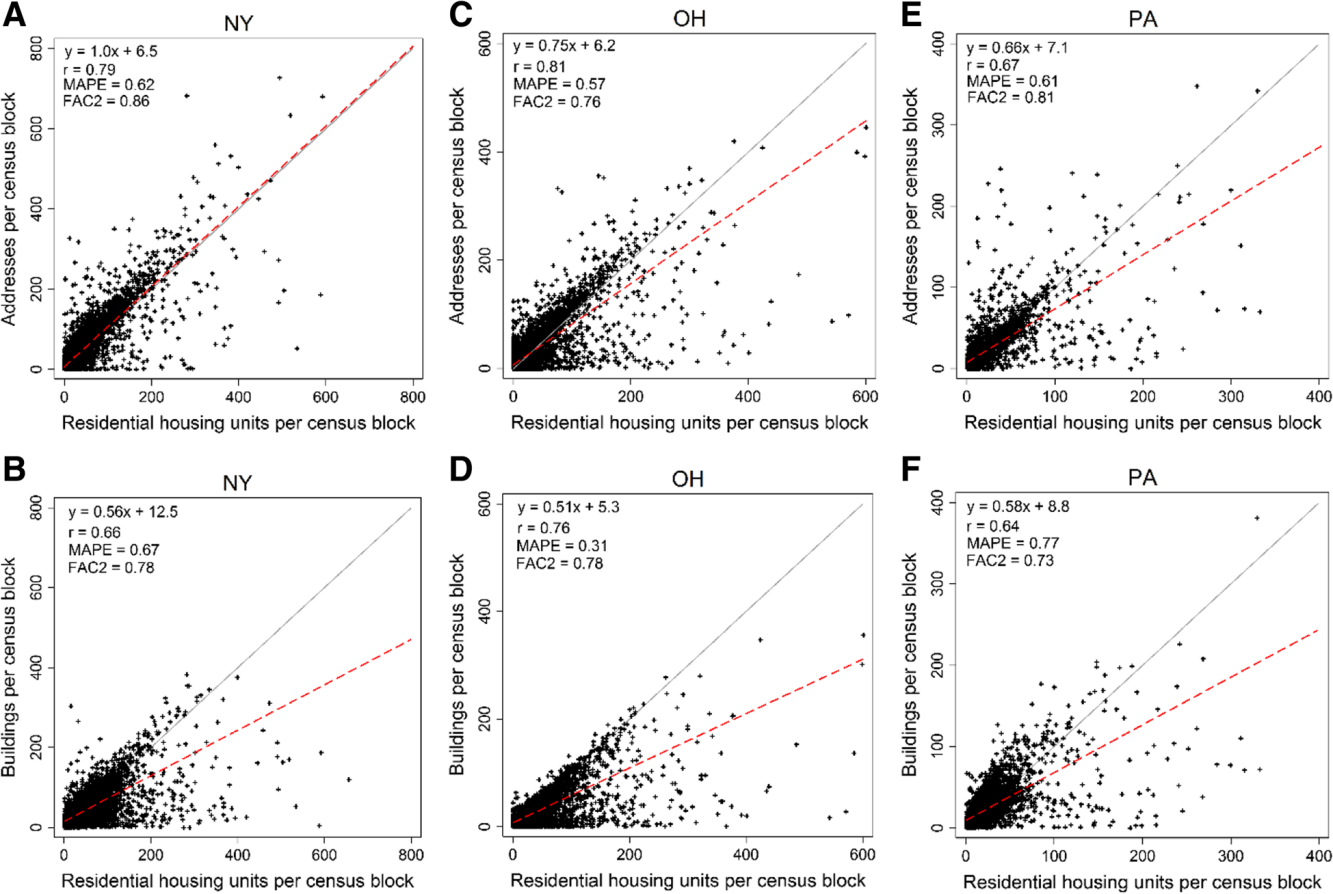
Total housing units per census blocks provided by U.S. Census data vs. residentially-censored addresses (top row) and residentially censored buildings (bottom row) across two selected counties for NY, OH, and PA. Panels **a** and **b** compare addresses and buildings to census RHUs for NY, panels **c** and **d** compare addresses and buildings to census RHUs for OH, and panels **e** and **f** compare adresses and buildings to census RHUs for PA.  One-to-one line is in grey, and each point represents a count within a census block

Address and building location data availability varied by state and by county (See Additional file 1: Table S5 for original sources). Because address and building data were not available for all areas, the assumption that address/building data equal counts of census housing units was tested only for two randomly-selected counties for each of NY, OH, and PA that contained complete address and building data. From these comparisons (Fig. 1), total residentially-sited addresses better predicted the total number of census block housing units compared to residentially-sited buildings (NY: *r* = .79 vs. 66; OH: *r* = .81 vs. 0.76; and, PA: *r* = 0.67 vs. 0.64, respectively). There also are differences in deviations from the one-to-one lines between address and building data as indicated by the slope of the liner fit lines, MAPE, and FAC2 values. While OH buildings did produce a smaller MAPE compared to addresses (0.31 vs. 0.57 - Fig. 1, panels c and d), apportioning populations to buildings would likely result in systematic PAR underestimations in part due its inability to capture multi-dwelling units (e.g., apartment complexes), which are more likely captured by address data. Moreover, the buildings data in OH represents only 84% of the total residential housing units, compared to the 114% of addresses vs. census housing units. Thus, for both data types, candidate RHU counts may disagree with census counts because of temporal misalignment; a portion of RHU location data is likely more recent and may account for changes subsequent to the 2010 census such as new housing development. Address data representativeness ranged at the county level from 2009 to 2018 (Additional file 1: Table S5).

Of addresses from OpenAddresses.io and the National Address Database (NAD) that intersected the 200 m^2^ well-areas, nearly 65% fell within built-up residential areas (exurban > urban > suburban) as per the Theobald [34] land use classification model (See Additional file 1: Table S2). Given the relatively high resolution of this land use model (30 × 30 m), this observation supports the assumption that these address data are sufficiently spatially accurate and sufficiently proxy for census-provided RHUs across various population morphologies (i.e., urban to rural). This observation also highlights the unique land use conflicts presented by UGS systems that were originally cited near city limits many decades prior and have since experienced population encroachment. Considering these results, we elected to use address location data over building data to proxy for RHUs for census population disaggregation across all six states for consistency, and RHUs henceforth refer to residentially-sited addresses. This decision is considered precautionary when considering the preference to overestimate PAR rather than underestimate. In acknowledging suboptimal address quality, RHUs located within setback well-areas (likely a higher risk class) were visually inspected via aerial imagery, and results are reported in Additional file 1: Table S3.

Uncapped RHU counts were greater than census block housing unit counts by 10, 8, and 19% for the two selected counties in PA, OH, and NY (Fig. 1), and the differences were significant (PA: *p* = <.0001; OH: *p* = <.0001; NY: *p* = <.0001). Under the assumption that overestimates could be due to inclusion of non-residential domiciles (i.e., empty parcels), rather than new RHU development following the 2010 census, we capped RHUs so that they do not exceed counts of housing units within an individual census block. Capped ABODE estimates no longer overestimated census-reported RHU counts for the selected counties, instead differences were − 13, − 12%, and − 4%, respectively. Notably, this adjustment only impacts total RHU and population enumerations, not intra-block RHU spatial heterogeneity. The capped and uncapped ABODE estimates in Table 2 therefore reflect the assumptions of potential address misclassification (capped) and potential new population/housing unit growth (uncapped).

**Table 2:** Population estimates and RHUs within 200 m of active UGS wells

State	Wells (% total) with at least one RHU	Uncapped RHUs	PAR (PPA)	PAR (ABODE)	PAR (Uncapped ABODE)	Well-level Wilcoxon Signed-Rank (Z) Ho: PPA = ABODE
CA	41 (12%)	400	1216	895	939	3.11^*^
MI	668 (28%)	2027	4740	4283	5158	6.64^***^
NY	362 (38%)	996	2495	2393	2691	3.81^**^
OH	1680 (51%)	12,014	27,593	29,184	31,273	1.11 (*p* = 0.2)
PA	577 (43%)	3709	4996	7421	9336	−5.99^***^
WV	488 (33%)	1791	2012	3093	3853	0.79^***^
Total	3816 (41%)	20,937	43,052	48,126	53,250	12.5^***^

^*^p = < 0.01; ^**^ *p* = < 0.001; ^***^p = < 0.0001

By either allocation method – ABODE or PPA – thousands of people and homes are likely within the proposed UGS Wellhead Safety Zone, and in some cases within state oil and gas well surface setback distances (See Table 4 and Additional file 1: Figure S2). By the PPA method, an estimated 43,052 people live within 200 m of the 9834 active UGS wells across the six states assessed (Table 2), while ABODE predicted between 48,126 and 53,250 (capped and uncapped) people within the same areas. Population differences between PPA and capped ABODE were significant at the well level (z = 12.5, *n* = 9834, *p* = < 0.0001, Wilcoxon signed-rank test). Notably, 6108 of the 200 m well-areas contained zero RHU’s; however, PPA only correctly predicted a zero population for 1082 of these areas, whereas ABODE correctly predicted zero for all 6108. In other words, PPA produced a false-positive non-zero population estimate for at least 5026 well-areas – over 50% of the total well-areas assessed. This difference can be understood through the underlying processes of the two methods – ABODE assumes residence in discrete spaces (i.e., physical structures), whereas PPA assumes some amount of population across continuous space. For ABODE, areas that do not contain an RHU do not contain people, whereas PPA does not check for RHUs when counting population. Thus, compared to PPA – in allocating identical census data to sub census block areas – ABODE’s discretionary allocation provides more precise PAR estimates by reducing PAR misclassification that occurs when PPA counts people in areas that do not contain RHUs.

In general, ABODE captured higher populations within 200 m of wells in OH, WV, and PA, with lower bound increases of 6% (1591), 54% (1081), and 48% (2425), respectively, compared to PPA estimates. Conversely, ABODE captured lower populations in CA (− 26% or 321). ABODE had similar estimates to PPA for NY and MI where PPA estimates fell within the bounds of capped and uncapped ABODE (see Table 2). Differences between methods were statistically significant for CA, MI, NY, WV, and PA, but not for OH (Table 2, and Additional file 1: Figure S3). A portion of the population difference observed in CA is likely driven by data quality via manual housing unit identification that likely underrepresented multi-dwelling units akin to using building data alone to represent RHUs. Population differences between methods were not mediated by the number of census blocks included or mean block area, and census block area was a poor predictor of census block populations (*R*^2^ = .02, RMSE = 67).

**Fig. 2:**
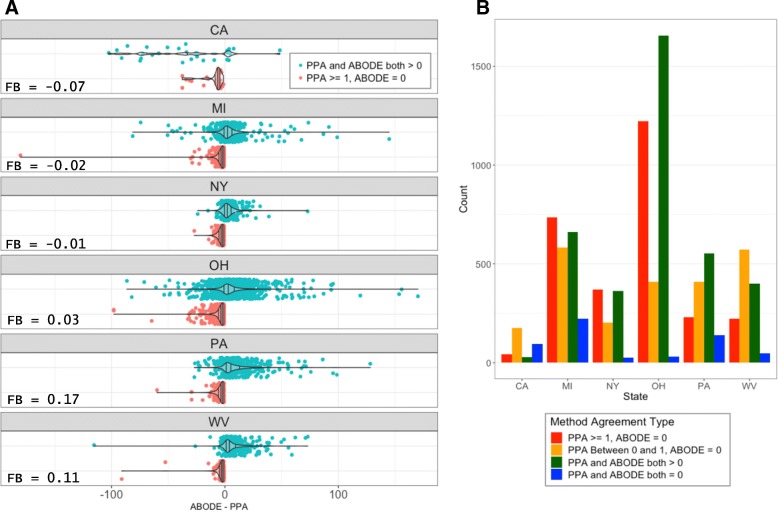
**a** Differences between population estimates (ABODE minus PPA) by state, differentiated by well-areas where both ABODE and PPA > 0 (blue), and where PPA predicted a population of at least 1 and ABODE = 0 (red). **b** frequency histogram of population method agreement type by state – counts of well-areas where PPA and ABODE both predicted positive populations (green), counts of well-areas were both methods predict zero (blue); and respective counts of confirmed false positives where PPA predicted between 0 and 1 (yellow) or greater than 1 (red) with ABODE predicting zero in both cases

A comparison of PPA vs. ABODE by state demonstrates the differences at the individual well level both across and within states (Fig. 2 & Additional file 1: Figure S3). In some cases, population estimates differed by more than 100 people between the two methods for individual 200 m-radius well-areas. Not surprisingly, the PPA method tends to overpredict populations when no physical housing units are present (Fig. 2, panel a - red markers). This was particularly prominent in MI where PPA predicted greater-than-zero estimates around 269 wells that did not contain any housing units within 200 m. Table 3 lists the top ten individual wells across the six states ranked by capped ABODE. Five of the top ten wells identified by ABODE did not make the top ten according to PPA – for three of these wells, PPA underestimated PAR by more than 100 people. Only 41 of CA’s 346 UGS wells contained an RHU within 200 m – the lowest percentage of the six states assessed. However, two wells in the Playa Del Rey field in CA ranked first and third respectively in the number of RHUs, and the estimated populations within 200 m indicate that UGS well-population relationships are not necessarily generalizable at the state or facility level. Nonetheless, UGS wells in OH, PA, MI, NY, and WV exhibited a much higher degree of land use conflicts in both magnitude and proximity compared to CA UGS wells.

**Table 3:** Top ten wells ranked by ABODE population at risk within 200 m

Storage field	State	RHUs	PAR (Capped ABODE)	PAR (PPA)	PAR difference	PPA PAR rank
1. Playa Del Rey	CA	150	341	375	−34	1
2. Zane Storage	OH	142	318	259	58	3
3. Playa Del Rey	CA	107	283	234	48	6
4. Medina	OH	88	257	87	170	109
5. Oakford	PA	146	255	127	128	43
6. Murrysville	PA	98	244	201	43	10
7. Stark-Summit	OH	95	236	176	59	16
8. Stark-Summit	OH	81	222	66	155	156
9. Stark-Summit	OH	95	221	162	59	20
10. Stark-Summit	OH	77	221	203	18	9

Of the 9384 UGS wells assessed across five states (there are no setback rules for CA wells), 444 wells associated with 69 storage facilities contained at least one RHU within its state’s regulated setback distance (see Table 4 and Additional file 1: Figure S2). This equated to a total of 905 visually verified RHUs (304 were manually removed) corresponding to an estimated 2171 people living within their home state’s setback. MI exhibited the most setback conflicts between wells and RHUs, but also promulgates the largest setback distance of the states included (300 ft). Similarly, over half of PA and WV facilities contained at least one well with a setback conflict.

**Table 4:** Population estimates and RHUs within state setbacks of active UGS wells

State	Applicable state setback distance (ft.)^a^	Facilities // wells with setback conflict	Visually verified RHUs within Setback	PAR (PPA)	PAR (ABODE)
CA	NA	NA	NA	NA	NA
MI	300	16 // 153	344	1277	532
NY	100	5 // 10	10	64	27
OH^a^	100	9 // 65	83	724	401
PA	200	25 // 124	296	550	792
WV	200	14 // 92	172	246	419
Total	–	69 // 444	905	2861	2171

^a^For additional information see Additional file 1: Table S1

**Fig. 3:**
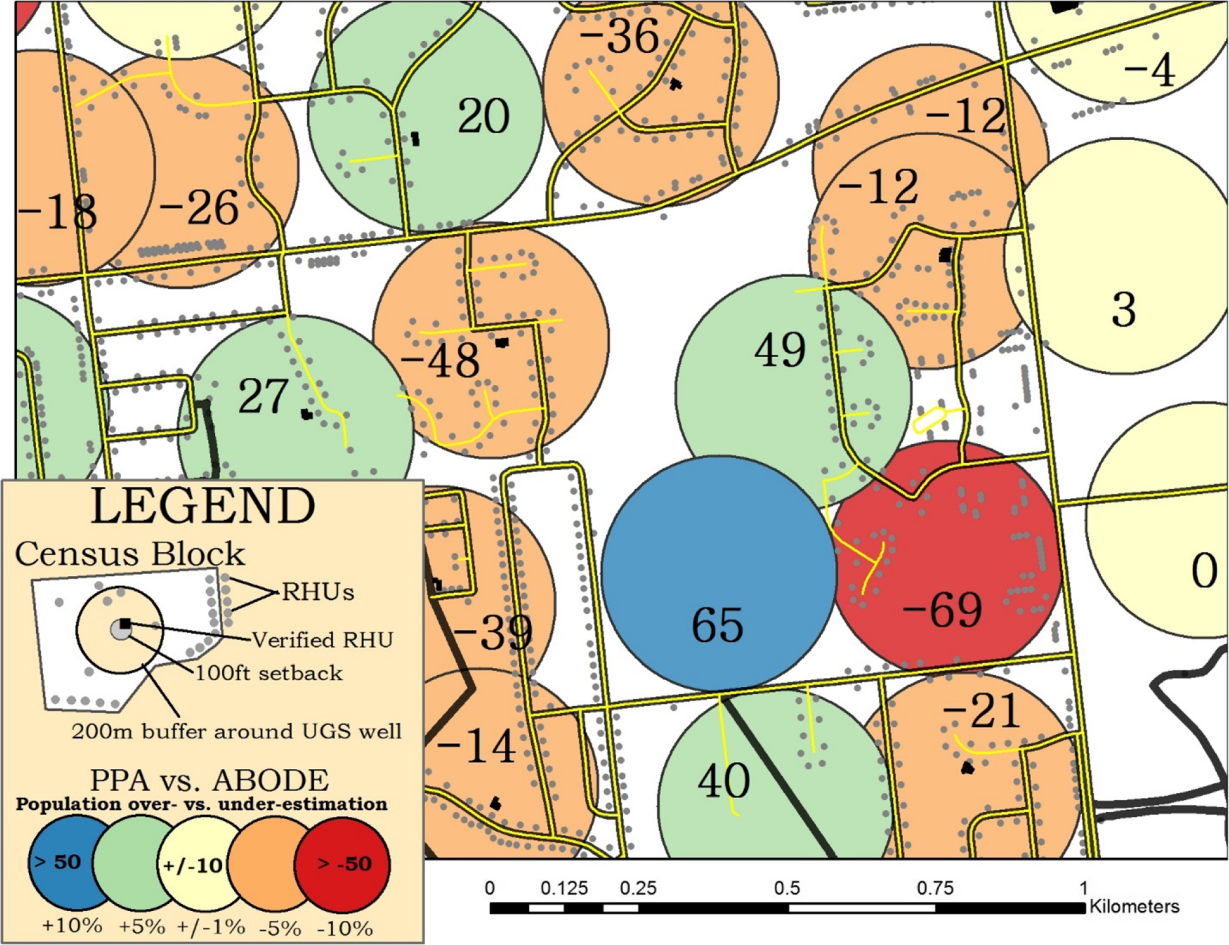
Active UGS wells with 200 m^2^ well-areas (multi-colored) within focused area of Stark County, OH. Black lines represent census block boundaries, yellow lines represent roadways, grey points represent RHUs, and black symbols represent visually verified RHUs within the 100 ft. setback well-area. Colors of the 200 m radial buffer distances represent over- and under-estimation of populations comparing PPA vs. uncapped ABODE. Blue circles indicate areas where PPA overestimated populations compared to ABODE by at least 50 people, or at least 10% of the entire county distribution. Red indicates where PPA underestimated by at least 50 people, or at least 10%, compared to ABODE

In line with 200 m results, PPA underestimated populations living within setback distances of wells in PA and WV; however, unlike PAR estimates at 200 m, PPA tended to overestimate ABODE at the smaller buffer areas for MI and OH. This suggests that PPA and other aerial weighting population allocation techniques are likely less reliable with decreasing search radii as the likelihood of capturing RHUs decreases with decreasing search radii. In other words, the distinction between areas of homogeneous density and discrete points of nighttime residential population is sharper at smaller spatial scales. Fig. 3 illustrates the principle that spatial heterogeneity of RHU’s can impact PAR estimates. For example, the dark blue 200 m well-area indicates that PPA overestimated the ABODE population by 65 (PPA estimated 65; ABODE estimated zero). Less than 400 m from this well location and in the same census block, the well-area in red was estimated to contain 81 people according to PPA – 69 fewer people than ABODE’s estimate of 151 people.

Figure 3 also demonstrates at sub-census block scales how PPA systematically breaks down compared to ABODE when the assumption of evenly distributed populations is violated – which it often is. Here we observe a patterned relationship between RHUs (grey points) and roadways (yellow) – that in many areas also double as census block boundaries (black lines). ABODE can account for this common pattern of homes (and residents) along roadways/census boundaries by first requiring RHUs in the search area prior to allocating population, whereas PPA treats all areas as if RHUs are evenly distributed within the block. Therefore, under this type of RHU/roadway pattern, PPA for small search areas that overlap with roadway/block boundaries will systematically underestimate the true population (i.e., true negative) and produce many false positives elsewhere. It is also important to note that census block sizes and shapes vary significantly across space and are not determined or controlled for by total population counts like census tracts, as indicated by the poor association between census block area and total population (*R*^2^ = 0.02; see Additional file 1: Figure S4).

## Discussion

The goals of this study were: 1) to develop a housing unit level population allocation method using appropriate address location- and building data spanning numerous states; 2) to apply these methods to estimate nighttime residential population at risk to UGS wells across six U.S. States; 3) to compare results to common aerial weighting estimates (e.g., PPA) at the sub-census block level to determine potential imprecision in similar PPA-PAR studies, and; 4) to utilize the curated RHU locations to assess the explicit spatial relationships between physical households and UGS wells in relation to the proposed UGS Wellhead Safety Zone and regulatory setbacks, where applicable.

Many studies have quantified populations at potentially higher risk of adverse health and safety effects due to their proximity to active oil and gas operations [35–40]. Most recently, Czolowski, Santoro [11] observed similar discrepancies across PAR proximity studies stemming primarily from inconsistent inclusion criteria for wells and ancillary infrastructure. However their national PAR estimates would likely not vary much vs. ABODE due to the relatively large search radii (one mile) that effectively reduces the total surface areas around wells that come in contact with non-whole census block areas – the interstitial areas where ABODE treats RHUs differently than PPA. McKenzie, Allshouse [29] applied a similar housing unit population allocation to assess population dynamics in Colorado using third-party real estate addresses geocoded with Google Maps. Our methods differed by using residentially-censored and publicly-available address location data with a persons-per-household estimate from the residing census block, as opposed to an aggregate multi-county average. Our study also further explored the implications of using discrete household geographic information to assess PAR using ABODE, particularly in comparison to PPA techniques and in relation to very small search areas such as state setbacks spanning 100-300 ft^2^.

A major limitation of all similar distance-based studies is the availability and quality of oil and gas well data, which can vary across facility, state, and over time [41]. While we did perform a limited sensitivity on well locational accuracy, a true visual verification would require an onsite inspection. Nonetheless, the range of locational accuracies observed herein underscores the importance of accuracy of both RHU proxy data and the hazard of interest when attempting to estimate PAR.

Because population estimates rely on census data, the primary sources of error are attributable to correctly identifying RHUs – both in terms of misclassification and temporal misalignment. It is important to note these errors can independently impact the efficacy of ABODE which could be conflated when comparing to other methods such as PPA. While we attempted to characterize potential for misclassification of RHUs, it is much more difficult to apportion errors to potential temporal misalignment between population data (2010), and address data that ranged between 2009 and 2018 (See Additional file 1: Table S5). Residentially-sited addresses from OpenAddresses.io and the partial National Address Database, while better than building data alone, only moderately predicted counts of census housing units, and misclassifications of occupied structures were observed in all states. Because estimates relying on building footprints alone tended to underpredict census housing units – but better captured physical domicile locations over address location – a better proxy for RHUs could be obtained by combining additional ancillary datasets such as parcel data or hybrid address/building data. The ongoing development of the U.S. Department of Transportation’s National Address Database stands to provide an authoritative source of accurate residence address location data nationwide that could support housing unit population mapping at larger scales, though much progress is still required [33]. Similarly, advances in remotely sensed object recognition and resolution refinement [42] have led to the first publicly available database of building footprint vector data for the entire U. S that could provide physical locations and verification to link to aforementioned address data [43].

Since residential structures do not move over time, whereas census aggregation units can, population estimates anchored to RHUs can attenuate the modifiable areal unit problem. However, some error remains in assuming an average person-per-household size. The number of residents in each household cannot be perfectly measured without labor intensive surveys; however household sizes could be further refined by using additional information about the residential housing units. A few studies have allocated populations based on building footprint area [22], and building volume estimated from aerial imagery data [20, 23], though these likely perform optimally for larger buildings in more urbanized areas. Estimates at the housing unit level also does not require census survey coverage if remote sensing building data are made available globally, and reasonable estimates of persons per household can be made. It is also important to note that population disaggregation methods that utilize census data are estimating the nighttime residential population- based on where people sleep. This ignores mobility of people, and the dynamic nature of populations in space and time, which also can introduce exposure and risk misclassification in epidemiological studies, and in other applications [44–46].

The major strength of ABODE is that it does not rely on the assumption of uniform spatial homogeneity of populations within an aerial unit. The formulation of ABODE better represents the real world – nighttime residence occurs in discrete locations (homes) rather than continuously across space. Violations of PPA’s assumption of spatial homogeneity were clearest in well-areas where zero visible RHU structures exist, yet PPA estimated a positive population (greater than zero) for over 50% of the total well-areas assessed. These estimates therefore can be confirmed as false positives (see Fig. 2). Based on our results across multiple sub-census block search areas, we expect PPA and similar aerial weighting techniques to lose precision and increase the likelihood of false positives with decreasing search radii. In tandem, PAR estimates backed by identification of RHUs provides an improved population density metric that carries a co-benefit of internal validation through the mutual inclusion of people and their physical dwellings. Such improved precision has relevance for estimating PAR to hazards with relatively short distance-based thresholds such as explosions, noise, air pollution, odors, sea level rise, radiation, and flooding.

Data presented herein provides new information regarding how close some homes and residents are to active UGS wells that are predominately located in suburban areas. The portion of RHUs identified within regulatory setback distances may result from several reasons. A significant portion of UGS wells likely predate any setback regulations for conventional wells (setback rules do not generally exist for other types of wells) due to their age (i.e., grandfathering). Other explanations include: 1) misclassification or spatial imprecision of UGS well and/or RHU, 2) a homeowner’s and/or regulatory agency’s consent to waive setback requirements for the drilling of new wells; and/or, 3) setback rules in the states assessed pertain only to placement of new wells in relation to existing buildings, not to the placement of homes in relation to existing wells (i.e., encroachment).

## Conclusion

Overall, our results indicate: 1) 65% of UGS wells across the six states assessed occupy residential urban and suburban areas with 41% containing at least one home within 200 m. 2) By resolving explicit spatial relationships between UGS wells and households, higher confidence estimates of populations at greatest risk can be made; 2) Bi-directional differences between ABODE and PPA estimates ranged from + 66% to − 26% at the state level and were even greater at the individual well which was primarily driven by the degree of RHU heterogeneity across space captured within 200 m well-areas; 3) PPA for small search areas that overlap with roadway/census block boundaries that are typically lined with RHUs will systematically underestimate the true population (i.e., true negative) and produce many false positives elsewhere; 4) PPA estimates are likely less reliable with decreasing search radii due to the decreasing probability of capturing actual RHUs; 5) by either method, OH exhibits a substantial degree of land use conflict between populations and UGS wells – more so than the other states assessed; and, 6) likely tens of thousands of homes and residents are located within the proposed UGS Wellhead Safety Zone – and in some cases within state oil and gas well surface setback distances – of active UGS wells.

## Additional file

Additional file 1: **SI Figure 1.** Methodological workflow and destination of results. **SI Table 1.** Methodological assumptions, anticipated biases, tests, data adjustments, and implications. **SI Table 2.** Number of individual land use parcels intersected by address points located within 200m of an active UGS well across the six states observed. Bold highlighted rows indicated land use types that contain addresses that were censored from inclusion as a residential housing unit. **SI Figure 2.** Frequency histograms of housing units within 200 m (657 ft) of active UGS well(s) by distance from well. Red lines indicate each state’s applicable regulatory surface setback distance for conventional oil and gas wells. The number displayed represents the number of visually verified housing units within the setback distance. The number displayed in the top right corner signifies total intersects including duplicates. **SI Figure 3.** PPA vs. ABODE population estimates of areas within 200m of active UGS wells. Dashed lines represent linear fits. Note each state plot contains unique scales. **SI Table 3.** UGS well and building counts within surface setbacks with visual verification results. **SI Table 4.** Legislative oil and gas setback restrictions for buildings. **SI Figure 4.** Total populations at the census block level vs. census block area for all six states assessed. **SI Figure 5.** Neighborhood level view of housing unit/address point and well data quality issues. **SI Table 5.** Address data original sources and publish date. OA = OpenAddresses.io, NAD = National Address Database. (DOCX 85961 kb)

## Data Availability

RHU data are available from corresponding author on reasonable request.

## Abbreviations

ABODE: Allocation by occupied domicile estimate
AUP: Aerial unit problem
BU: Built-up
CA: California
MI: Michigan
NAD: National Address Database
NAIP: National Agriculture Imagery Program
NY: New York
OH: Ohio
PA: Pennsylvania
PAR: Population at risk
PPA: Proportional population allocation
RHU: Residential housing unit
RMSE: Root mean square error
SD: Standard deviation
UGS: Underground natural gas storage
WV: West Virginia
