# Supplementary material for: Population allocation at the housing unit level: estimates around underground natural gas storage wells in PA, OH, NY, WV, MI, and CA
Source: Environ Health. 2019 Jul 8;18:58. doi: 10.1186/s12940-019-0497-z (PMC6613251; doi:10.1186/s12940-019-0497-z)
Supplement: Supplementary file 1 — SI Figure 1. Methodological workflow and destination of results. SI Table 1. Methodological assumptions, anticipated biases, tests, data adjustments, and implications. SI Table 2. Number of individual land use parcels intersected by address points located within 200m of an active UGS well across the six states observed. Bold highlighted rows indicated land use types that contain addresses that were censored from inclusion as a residential housing unit. SI Figure 2. Frequency histograms of housing units within 200 m (657 ft) of active UGS well(s) by distance from well. Red lines indicate each state’s applicable regulatory surface setback distance for conventional oil and gas wells. The number displayed represents the number of visually verified housing units within the setback distance. The number displayed in the top right corner signifies total intersects including duplicates. SI Figure 3. PPA vs. ABODE population estimates of areas within 200m of active UGS wells. Dashed lines represent linear fits. Note each state plot contains unique scales. SI Table 3. UGS well and building counts within surface setbacks with visual verification results. SI Table 4. Legislative oil and gas setback restrictions for buildings. SI Figure 4. Total populations at the census block level vs. census block area for all six states assessed. SI Figure 5. Neighborhood level view of housing unit/address point and well data quality issues. SI Table 5. Address data original sources and publish date. OA = OpenAddresses.io, NAD = National Address Database. (DOCX 85961 kb) [file 12940_2019_497_MOESM1_ESM.docx]

**Population allocation at the housing unit level: Estimates around underground natural gas storage wells in PA, OH, NY, WV, MI, and CA**

Drew R. Michanowicz^1^, Samuel R. Williams^1,2^, Jonathan J. Buonocore^1^, Sebastian T. Rowland^3^, Katherine E. Konschnik^4^, Shaun A. Goho^5^, Aaron S. Bernstein^1,6^

^1^ Center for Climate, Health and the Global Environment, Harvard T.H. Chan School of Public Health, Boston, MA 02215, United States

^2^ Department of Environmental Health, Boston University, Boston, MA, 02215, United States

^3^ Department of Environmental Health Sciences, Columbia University, New York City, NY 10027, United States

^4^ Nicholas Institute for Environmental Solutions, Duke University, Durham, NC 27708, United States

^5^ Emmett Environmental Law & Policy Clinic, Harvard Law School, Cambridge, MA 02138, United States

^6^ Division of General Medicine, Boston Children’s Hospital, Boston, MA 02115, United States

Address correspondence to D. Michanowicz, 401 Park Drive, Landmark Center 4^th^ floor west suite 415E, Boston, MA, 02215 USA. Telephone: (814) 934-5624, Email: [michanow@hsph.harvard.edu](mailto:michanow@hsph.harvard.edu),

A methodological workflow in shown in SI Fig. 1. The boxes represent a data source or important step that alters or manipulates that data resulting in an output as indicated by the arrows and ovals. Locations of results herein are also indicated including sensitivity tests performed on the UGS wells (Table 4), and the address location data (Fig. 5). Notably the housing units visually verified at the respective state setback distances were utilized as opposed to the raw residential housing units to produce ABODE population estimates as indicated below.

**
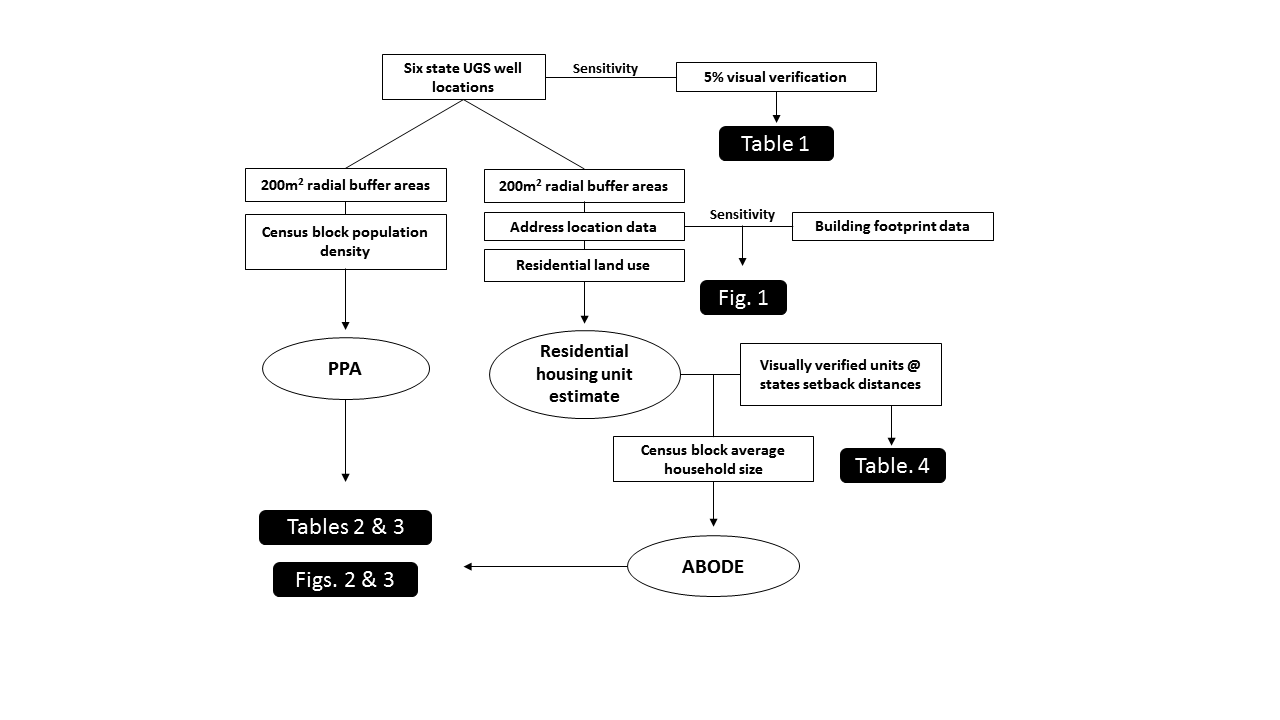
**

**SI Figure 1** Methodological workflow and destinations of results.

**SI Table 1** Methodological assumptions, anticipated biases, tests, data adjustments, and implications

| **Assumption** | **Anticipated direction of bias if assumption is false** | **Testable** | **Results of test** | **Data Adjustment** | **Implication of Test/Adjustment** |
| --- | --- | --- | --- | --- | --- |
| US Census population counts represent true underlying distribution | Likely underestimation of true distribution | NA | NA | NA | NA |
| Address points proxy only for residential housing units | Overestimation if non-residential address/building; underestimation if fail to represent multi-housing units | Location of address points intersected with high-resolution geographic land use data | Addresses point locations intersecting with various land use types shown in SI Table 2 | Address points intersecting with industrial and commercial land use types were removed | reduces likelihood of overestimation of residential housing units and population. Likely additional misclassification unaccounted for |
| Address points proxy for housing units by a 1:1 relationship (i.e., counts) | Over- and underestimation | 1:1 plots of address points and housing units summed at the census block level | In total, censored address points overpredicted housing units; bi-directional bias observed at smaller spatial scales available (state, county, census block, individual well) | Raw data presented, and artificially capped address points at the housing unit total at the census block | Raw (uncapped) estimates overestimate census housing unit counts and overestimate populations in some census blocks. |
| Address coordinate points are spatially accurate in representing physical structure | Over- and underestimation | Partial visual assessment performed within state surface setback areas | A portion of address points were removed as shown in SI Table 3. | Address points not visually verified in setbacks were removed for population estimates at setback and 200m^2^ | Likely overestimation of address points as housing units and population estimates at the full 200m. Address point missingness unknown |
| Well locations are spatially accurate | Over- and underestimation of setback violations and populations | 5% of wells were randomly selected and visually assessed | Varied by state; Median error distance range = 10.9 - 82.8ft. (See SI Table 2) | NA | NA |
| Address points spatially underrepresent building footprints | Over- and underestimation of setback violations and populations | Compared likelihood of intersection inclusion for counties with both addresses and footprints | On average, addresses underestimate inclusion in 200m buffer by 18% | NA | Test indicates that building and population estimates likely underestimate underlying distributions |
| Average household size equals true population | Over- and underestimation of populations | NA | NA | NA | NA |

**Land use data**

National land use classification schemes and data were obtained from Theobald [24]. Briefly, these data include a comprehensive land use classification of the conterminous US at 30 m^2^ resolution utilizing nearly two-dozen publicly-available national spatial datasets – including census housing and satellite data. In total 79 land use classes were determined in five main land use groups: built-up, production, recreation, conservation, and water. To censor non-residential address points, address points that intersected all built-up industrial, and built-up commercial zoned areas. 816 address points within the 235 individual non-residential parcels shown in SI Table 2 were censored.

**SI. Table 2** number of individual land use parcels intersected by address points located within 200m of an active UGS well across the six states observed. Bold highlighted rows indicated land use types that contain addresses that were censored from inclusion as a residential housing unit

| **Land Use Type** | **Parcel Count** |
| --- | --- |
| BU Res. Exurban | 4224 |
| BU Res. Urban | 3919 |
| BU Res. Suburban | 3034 |
| BU Res. Rural | 2635 |
| Prod Pastureland | 1104 |
| Prod Grazed | 831 |
| Prod Row crops | 486 |
| BU Trans. Highways/railways | 455 |
| **BU Comm. Retail/shopping** | **125** |
| Water Wetlands | 68 |
| **BU Comm. Office** | **64** |
| Rec Dev. Resort/Ski area | 55 |
| **BU Ind. Factory, plant** | **46** |
| Cons. Public-limited Corps Engineers | 35 |
| Rec Dev. undifferentiated | 29 |
| Water Nat. Lake | 25 |
| BU Misc | 25 |
| BU Trans. Undeveloped | 23 |
| Cons. Public Nature Reserve | 19 |
| **BU Inst. Medical** | **18** |
| **BU Inst. Schools (undeveloped)** | **14** |
| Rec Dev. Golf course | 14 |
| Water Hum. Reservoir | 12 |
| Cons. Public Wildlife habitat | 12 |
| Water River | 9 |
| BU Res. dense urban | 8 |
| Water Nat. Playa | 7 |
| BU Trans. Airports | 7 |
| Rec. Nat. park | 7 |
| Prod orchards | 6 |
| Prod Timber | 6 |
| **BU Inst. Government** | **4** |
| **BU Inst. Schools (developed)** | **3** |
| Prod Cropland (general) | 3 |
| **BU Ind. Landfill waste** | **2** |
| Rec Dev. Urban park | 1 |


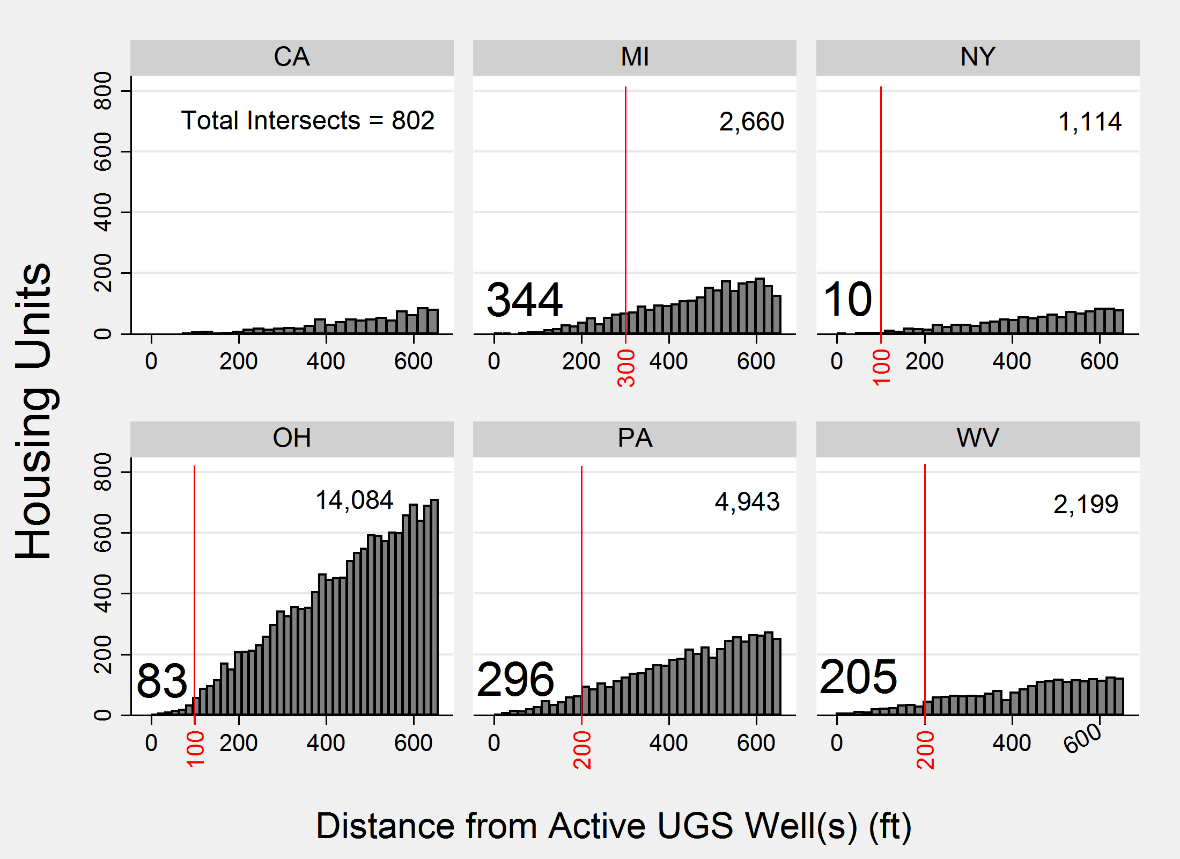


**SI Figure 2** Frequency histograms of housing units within 200m^2^ (657 ft) of active UGS well(s) by distance from well. Red lines indicate each state’s applicable regulatory surface setback

distance for conventional oil and gas wells. The number displayed represents the number of visually verified housing units within the setback distance. The number displayed in the top right corner signifies total intersects including duplicates.


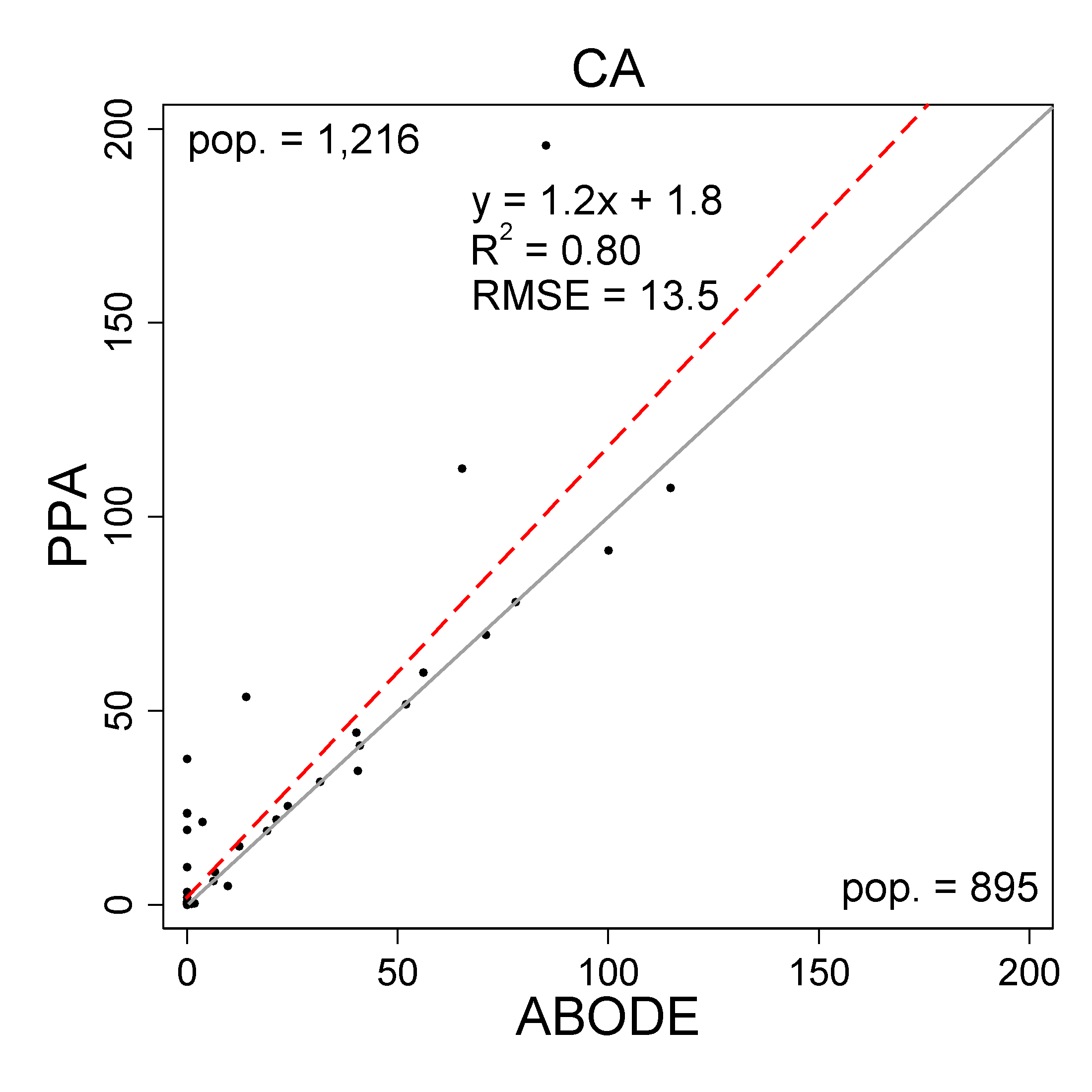

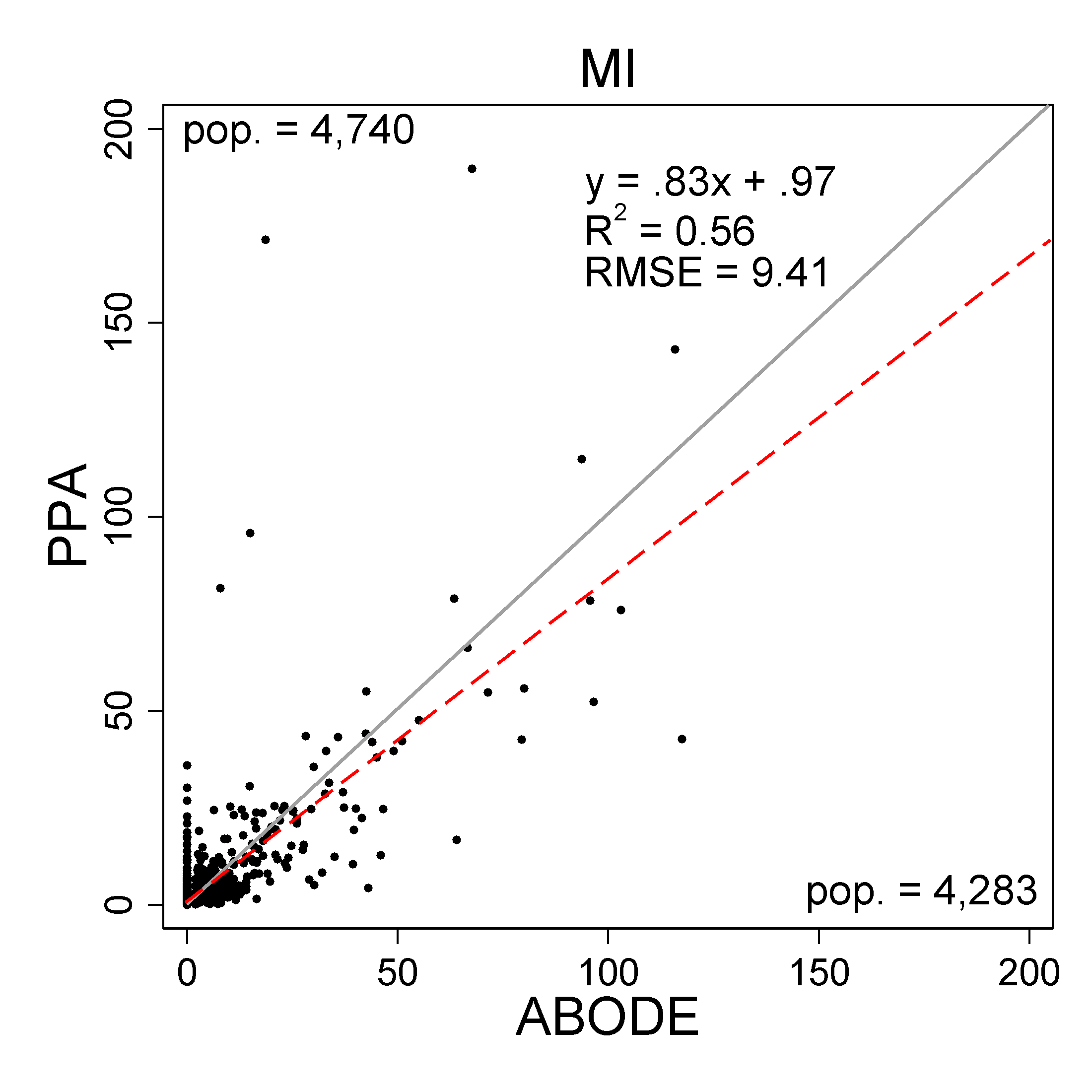

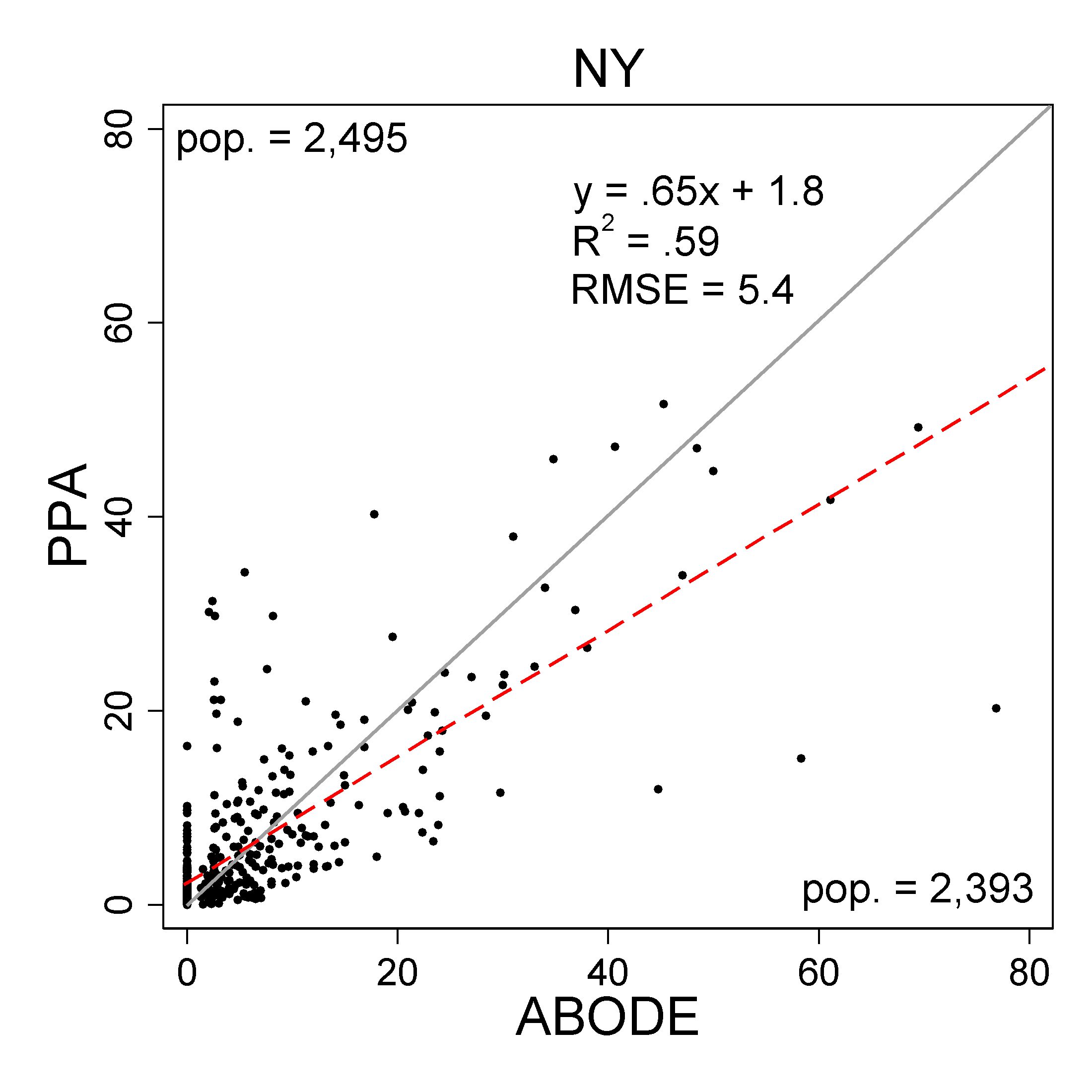


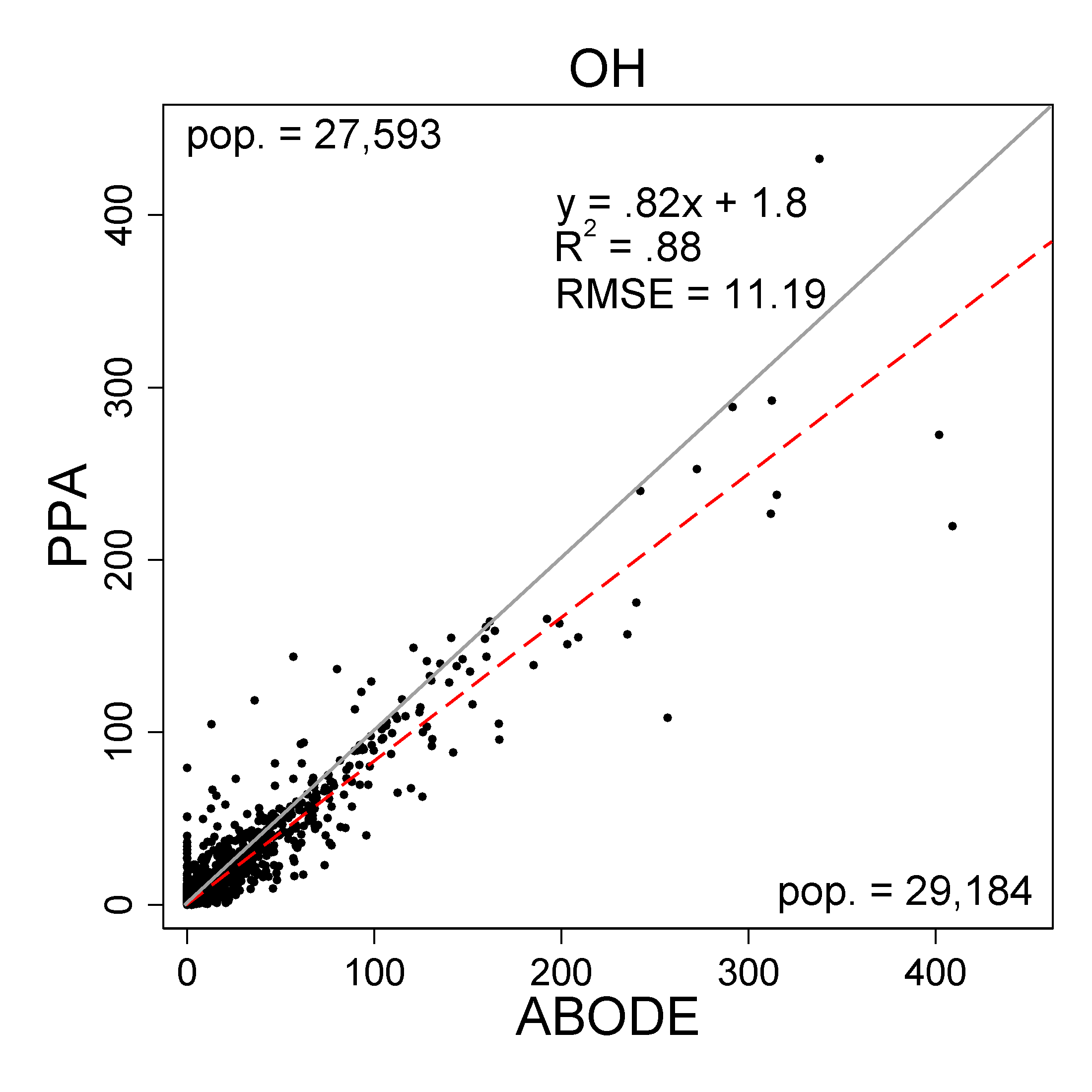

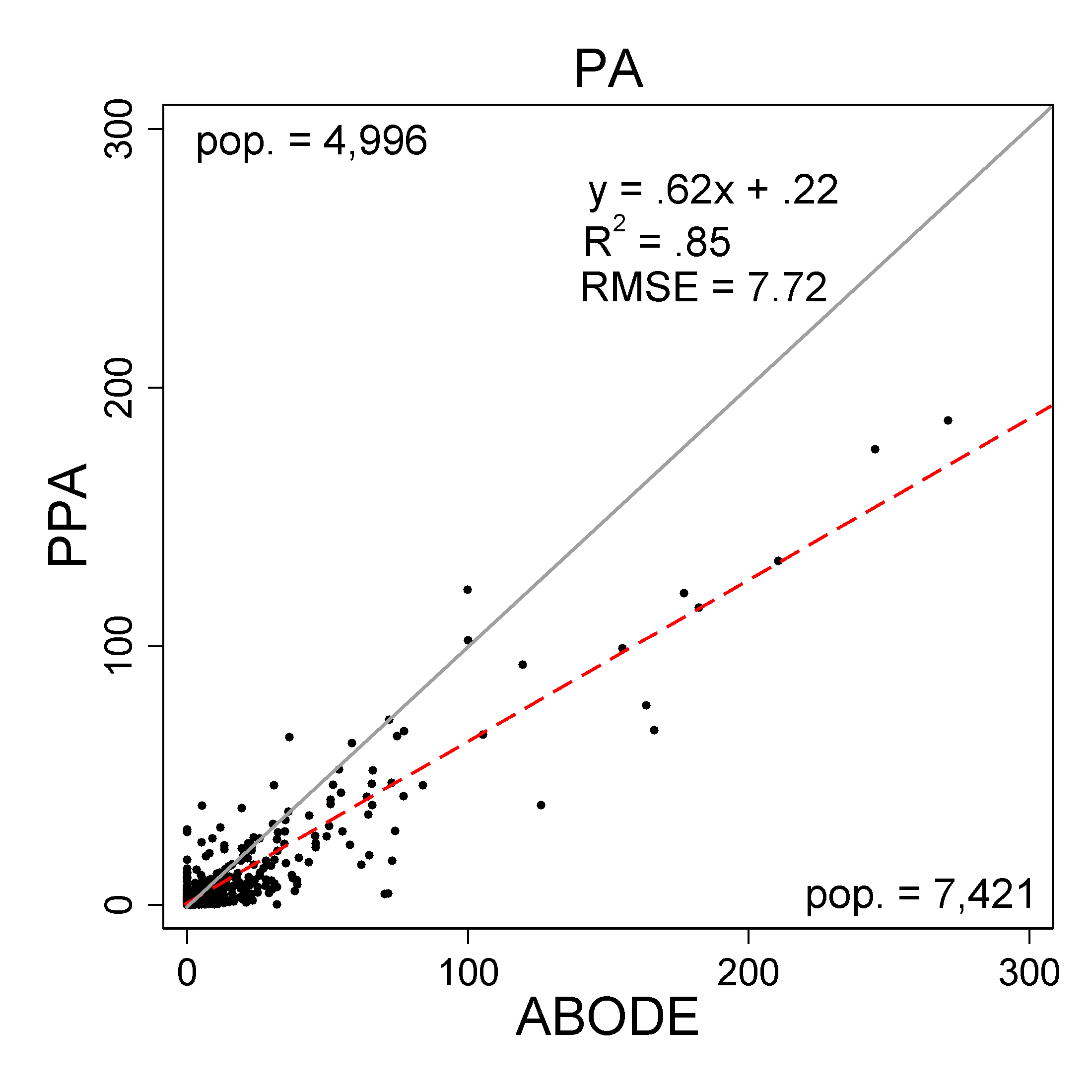

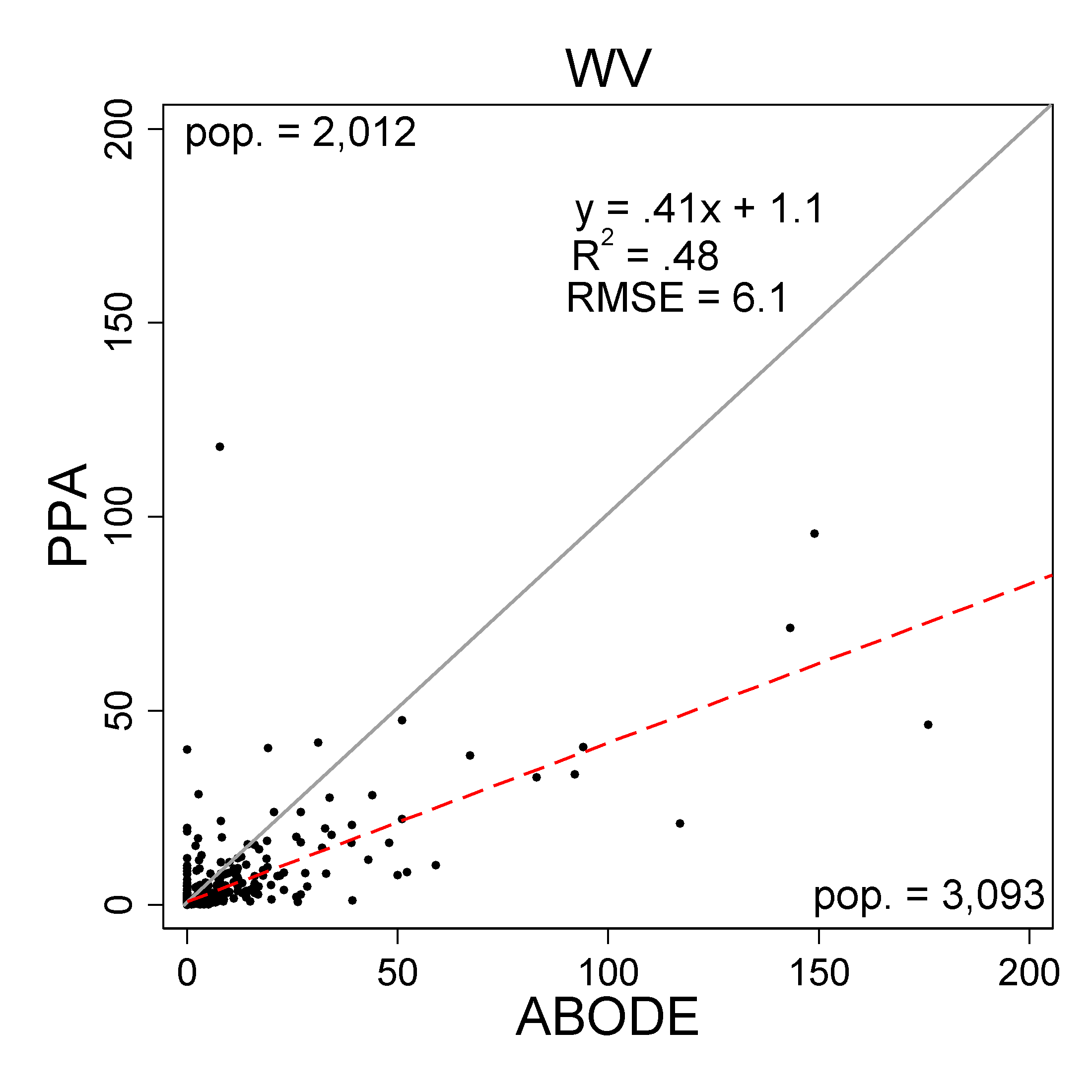


**Figure 3** PPA vs. ABODE population estimates of areas within 200m of active UGS wells. Dashed lines represent linear fits. Note each state plot contains unique scales.

**SI Table 3** UGS well and building counts within surface setbacks with visual verification results

| State | Building setback distance (ft.) | Address points within setback | Visual verification distance | Setback addresses removed | Visually verified housing units | Facilities (wells) with setback conflict |
| --- | --- | --- | --- | --- | --- | --- |
| CA | NA | NA | 200m | NA | NA | (NA) |
| MI | 300 | 419 | 300ft. | 75 | 344 | 16 (153) |
| NY | 100 | 12 | 100ft. | 2 | 10 | 5 (10) |
| OH | 100 | 159 | 100ft. | 76 | 83 | 9 (65) |
| PA | 200 | 356 | 200ft. | 60 | 296 | 25 (124) |
| WV | 200 | 235 | 200ft. | 91 | 172 | 14 (92) |
| **Total** | - | **1,181** | - | **304** | **905** | **69 (444)** |

**SI Table 4** Legislative oil and gas setback restrictions for buildings

| **State** | **Well Setback**  **Distance from Building (ft)** | **Detail and Regulatory Statute** |
| --- | --- | --- |
| CA | NA | California does not regulate the distance between wells and buildings, but does regulate the distance between wells and public roadways. |
| OH | 100 | The surface location of a new well…shall not be within one hundred fifty feet of an occupied dwelling that is located in an urbanized area unless…the chief of the division of oil and gas resources management approves the written consent of the land owner. Ohio R.C. § 1509.021(A). For areas that are not urbanized areas, the surface location of a new well shall not be within one hundred feet of an occupied private dwelling or of a public building that may be used as a place of assembly, education, entertainment, lodging, trade, manufacture, repair, storage, or occupancy by the public  Ohio R.C. § 1509.021(H). |
| MI | 300 | “Wells must be at least 300 feet from existing structures used for public or private occupancy.” Surface facilities may be located closer than 300 feet from…existing structures used for public or private occupancy under either of the following conditions: (a) Upon presentation to the supervisor of mineral wells of a written consent signed by the owner or owners of all…existing structures used for public or private occupancy. (b) After…the supervisor of mineral wells determines that the proposed surface facility location will prevent waste, protect environmental values, and not compromise public safety. (4) The supervisor of mineral wells or authorized representative of the supervisor of mineral wells shall be notified within 24 hours of emergency repairs to existing surface facilities that substantially modify the facility or piping. Mich. Admin. Code R. §299.2441 |
| PA | 200 | “Wells may not be drilled within 200 feet, or, in the case of an unconventional gas well, 500 feet, measured horizontally from the vertical well bore to a building without written consent of the owner of the building or water well.” “The variance shall include additional terms and conditions required by the department to ensure safety and protection of affected persons and property, including insurance, bonding, indemnification and technical requirements.” 58 Pa. Stat. §3215(a). |
| NY | 100 | “No well shall be located nearer than 100 feet from any inhabited private dwelling house without written consent of the owner; nearer than 150 feet from any public building or area which may be used as a place of resort, assembly, education, entertainment, lodging, trade, manufacture, repair, storage, traffic or occupancy by the public. This regulation, which is adopted in the interest of public safety, does not apply to a building or structure which is incident to agricultural use of the land on which it is located, unless such building is used as a private dwelling house or in the business of retail trade. 6 CRR-NY §553.2 |
| WV | 200 | W. Va. Code Ann. § 22-6-21 provides that: “No oil or gas well shall be drilled nearer than two hundred feet from an existing water well or dwelling without first obtaining the written consent of the owner of such water well or dwelling.”  The regulations indicate that an application for a permit for an “underground storage well” must include “[i]f applicable, the consent required by W. Va. Code § 22-6-21.”  W. Va. Code R. 35-4-5.  “Underground storage well” is defined as “a gas well subject to the provisions of W. Va. Code § 22-9-1, et seq.”—an article called “Underground Gas Storage Reservoirs.”  W. Va. Code R. 35-4-2. |


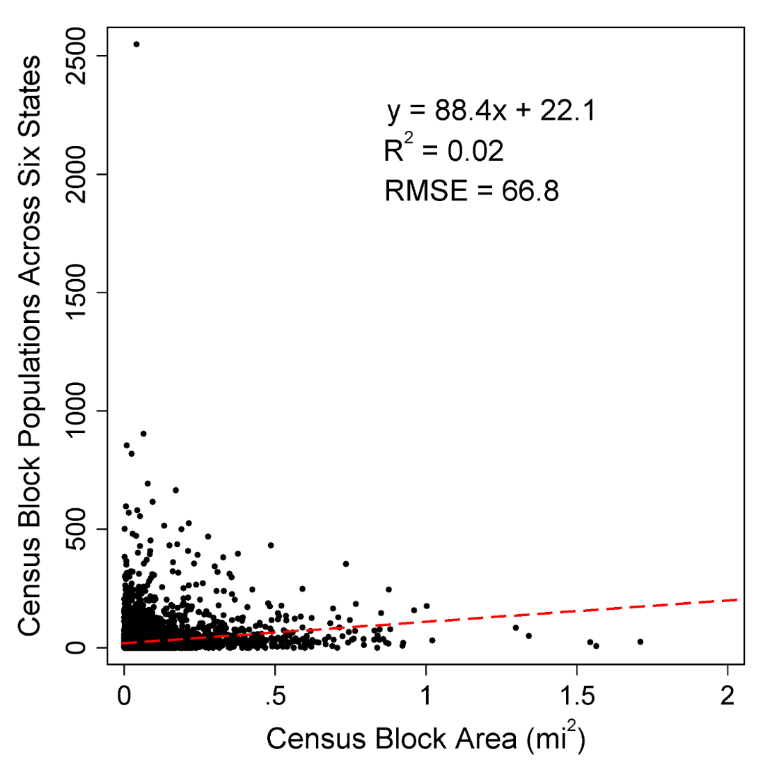


**SI Figure 4** Total populations at the census block level vs. census block area for all six states assessed.


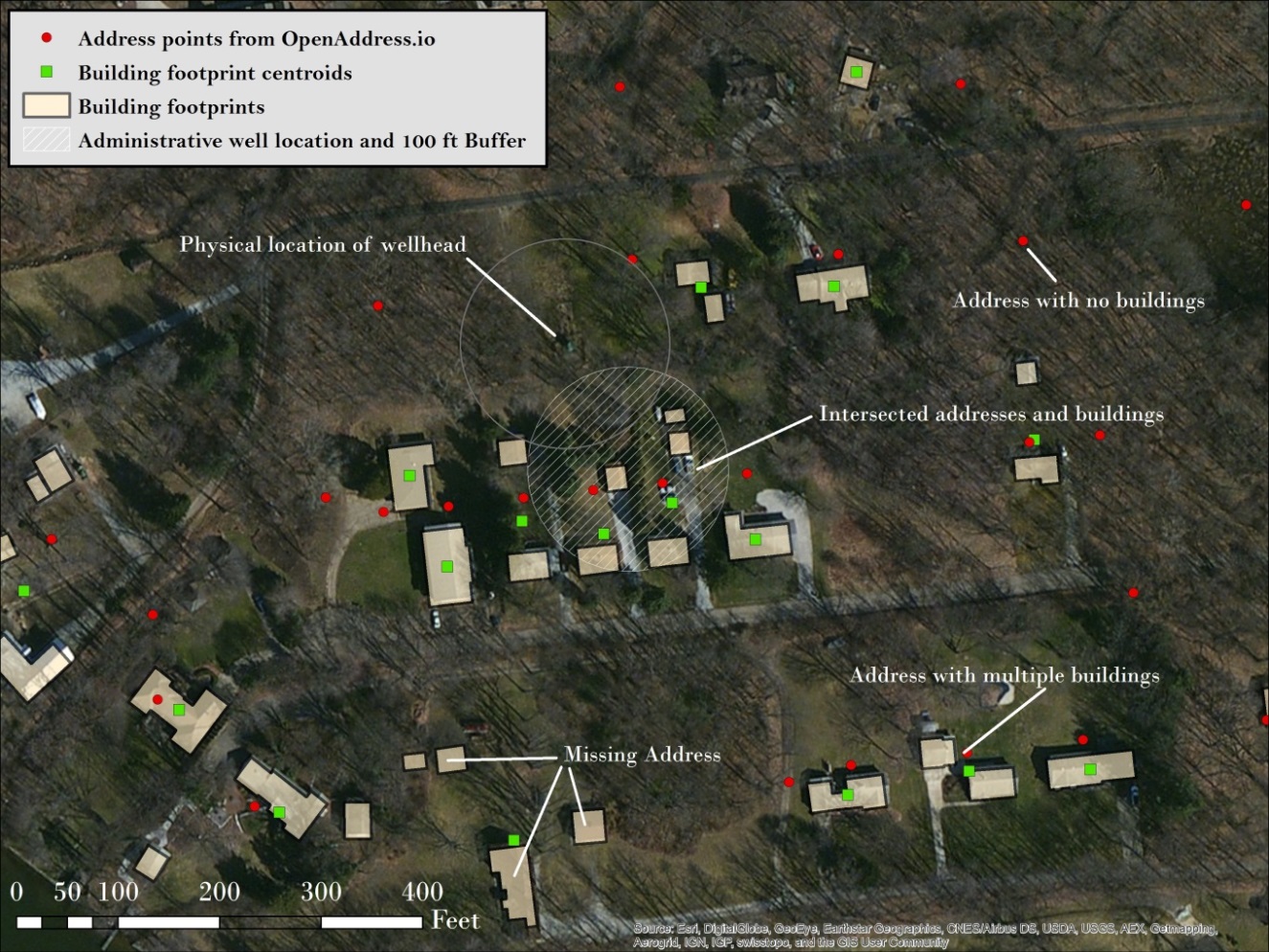


**SI Figure 5** Neighborhood level view of housing unit/address point and well data quality issues

SI Fig. 4 provides an illustrative example of some of the well location and address/building data quality issues described herein. Notably, the “address with no buildings” points indicate the type of candidate RHU address points that would removed if they fell within grey crosshatched 100ft setback area resulting in the “visually verified RHUs within Setback” shown in Table 4. The effect of inaccurate well locations can also be observed in SI Fig. 4, where the administrative well location places a well approximately 40m south of the location as determined by aerial imagery. This spatial misalignment can result in substantial errors in estimating nearby buildings and populations, though this bias likely does not have a systematic directionality.

**SI Table 5. Address data original sources and publish date. OA = OpenAddresses.io, NAD = National Address Database**

| **State** | **County** | **Aggregator** | **Data Source** | **Date Published** | **Manual Add? Y/N** |
| --- | --- | --- | --- | --- | --- |
| CA | Butte | NA | Manual | NA | Y |
| CA | Colusa | NA | Manual | NA | Y |
| CA | Contra Costa | NA | Manual | NA | Y |
| CA | Los Angeles | NA | Manual | NA | Y |
| CA | Madera | NA | Manual | NA | Y |
| CA | San Joaquin | NA | Manual | NA | Y |
| CA | Santa Barbara | NA | Manual | NA | Y |
| CA | Solano | NA | Manual | NA | Y |
| CA | Yolo | NA | Manual | NA | Y |
| MI | Allegan | OA | https://gis.allegancounty.org/gis/rest/services/ParcelViewer2/PV2AdressesV2_1/MapServer | 2018 | Y |
| MI | Barry | NA | Manual | NA | Y |
| MI | Calhoun | NA | Manual | NA | Y |
| MI | Clare | NA | Manual | NA | Y |
| MI | Eaton | OA | https://services2.arcgis.com/c9l1e4fKpsCnqD7H/arcgis/rest/services/Site_Address_Point/FeatureServer/0 | 2019 | Y |
| MI | Ingham | NA | Manual | NA | Y |
| MI | Kalkaska | NA | Manual | NA | Y |
| MI | Lake | NA | Manual | NA | Y |
| MI | Livingston | NA | Manual | NA | Y |
| MI | Macomb | OA | https://github.com/openaddresses/openaddresses/blob/master/sources/us/mi/macomb.json | 2017 | Y |
| MI | Mecosta | NA | Manual | NA | Y |
| MI | Missaukee | NA | Manual | NA | Y |
| MI | Montcalm | NA | Manual | NA | Y |
| MI | Montmorency | NA | Manual | NA | Y |
| MI | Newaygo | OA | http://arcgisweb.countyofnewaygo.com:6080/arcgis/rest/services/WebMaps/NewaygoCounty/MapServer/2 | NA | Y |
| MI | Oakland | OA | https://gisservices.oakgov.com/arcgis/rest/services/Enterprise/EnterpriseOpenParcelDataMapService/MapServer/0 | NA | Y |
| MI | Osceola | NA | Manual | NA | Y |
| MI | Otsego | NA | Manual | NA | Y |
| MI | Ottawa | OA | http://gis.co.ottawa.mi.us/gisweb/rest/services/layers/Parcel/MapServer/1 | NA | Y |
| MI | St. Clair | OA | https://maps.stclaircounty.org/Html5/index.html?viewer=Public_Pcls | 2019 | Y |
| MI | Washtenaw | NA | Manual | NA | Y |
| NY | Allegany | NAD | New York State GIS Program Office | 2015-2017 | N |
| NY | Cattaraugus | NAD | New York State GIS Program Office | 2015-2017 | N |
| NY | Chautauqua | NAD | New York State GIS Program Office | 2015-2017 | N |
| NY | Erie | NAD | New York State GIS Program Office | 2015-2017 | N |
| NY | Onondaga | NAD | New York State GIS Program Office | 2015-2017 | N |
| NY | Schuyler | NAD | New York State GIS Program Office | 2015-2017 | N |
| NY | Steuben | NAD | New York State GIS Program Office | 2015-2017 | N |
| NY | Tioga | NAD | New York State GIS Program Office | 2015-2017 | N |
| NY | Wyoming | NAD | New York State GIS Program Office | 2015-2017 | N |
| NY | Yates | NAD | New York State GIS Program Office | 2015-2017 | N |
| OH | Ashland | Both | http://gis3.oit.ohio.gov/LBRS/_downloads/ASH_ADDS.zip | 12/1/2016 | N |
| OH | Columbiana | Both | http://gis3.oit.ohio.gov/LBRS/_downloads/COL_ADDS.zip | 11/8/2017 | N |
| OH | Coshocton | Both | http://gis3.oit.ohio.gov/LBRS/_downloads/COS_ADDS.zip | 11/8/2017 | N |
| OH | Fairfield | Both | http://gis3.oit.ohio.gov/LBRS/_downloads/FAI_ADDS.zip | 11/8/2017 | N |
| OH | Guernsey | Both | http://gis3.oit.ohio.gov/LBRS/_downloads/GUE_ADDS.zip | 11/8/2017 | N |
| OH | Hocking | Both | http://gis3.oit.ohio.gov/LBRS/_downloads/HOL_ADDS.zip | 11/8/2017 | N |
| OH | Holmes | Both | http://gis3.oit.ohio.gov/LBRS/_downloads/HOL_ADDS.zip | 12/1/2016 | N |
| OH | Lorain | Both | http://gis3.oit.ohio.gov/LBRS/_downloads/LUC_ADDS.zip | 11/8/2017 | N |
| OH | Medina | NA | Manual | NA | Y |
| OH | Muskingum | Both | http://gis3.oit.ohio.gov/LBRS/_downloads/MUS_ADDS.zip | 4/25/2018 | N |
| OH | Perry | Both | http://gis3.oit.ohio.gov/LBRS/_downloads/PER_ADDS.zip | 12/1/2016 | N |
| OH | Richland | Both | http://gis3.oit.ohio.gov/LBRS/_downloads/RIC_ADDS.zip | 12/1/2016 | N |
| OH | Stark | Both | http://gis3.oit.ohio.gov/LBRS/_downloads/STA_ADDS.zip | 11/8/2017 | N |
| OH | Summit | Both | http://gis3.oit.ohio.gov/LBRS/_downloads/SUM_ADDS.zip | 11/8/2017 | N |
| OH | Vinton | Both | http://gis3.oit.ohio.gov/LBRS/_downloads/VIN_ADDS.zip | 11/8/2017 | N |
| OH | Wayne | Both | http://gis3.oit.ohio.gov/LBRS/_downloads/WAY_ADDS.zip | 11/8/2017 | N |
| PA | Allegheny | OA | http://gisdata.alleghenycounty.us/arcgis/rest/services/Addressing/Addressing_AddressPoints/MapServer | 1/9/2018 | N |
| PA | Armstrong | OA | https://raw.githubusercontent.com/aaronpdennis/pa-county-addresses/master/data/ARMSTRONG/ARMSTRONG_addresses.csv | 1/1/2009 | N |
| PA | Beaver | OA | http://bkrgis.bakerprojects.com/ArcGIS/rest/services/BeaverCounty/BeaverCounty_Addresses/MapServer/0 | NA | N |
| PA | Bedford | OA | https://services2.arcgis.com/tXFMtuwRfEDEFdnG/ArcGIS/rest/services/Bedford_County_Addresses/FeatureServer/0 | 10/1/2018 | N |
| PA | Butler | OA | http://maps.co.butler.pa.us/arcgis/rest/services/ButlerCounty/ButlerMapService/MapServer/1 | NA | N |
| PA | Clearfield | OA | http://gis.clearfieldco.org/arcgis/rest/services/Web_Map/MapServer/1 | NA | N |
| PA | Clinton | OA | http://maps.clintoncountypa.com/arcgis/rest/services/Testing/STRUCTURES/MapServer/0 | NA | N |
| PA | Elk | OA | https://github.com/aaronpdennis/pa-county-addresses/raw/master/data/ELK/ELK_addresses.csv | 12/1/2009 | N |
| PA | Erie | OA | https://github.com/aaronpdennis/pa-county-addresses/raw/master/data/ERIE/ERIE_addresses.csv | 12/1/2009 | N |
| PA | Fayette | OA | http://fayettemaps.rba.com:8082/geoserver/fayette/ows?service=WFS&version=1.0.0&request=GetFeature&typename=fayette:ParcelsPolygon&outputFormat=json | 12/13/2017 | N |
| PA | Forest | OA | http://web1.mobile311.com/arcgis/rest/services/Pennsylvania/ForestCountyPa/MapServer/0 | 4/4/2017 | N |
| PA | Greene | OA | http://54.235.209.242/arcgis/rest/services/GreeneCounty_PA/GreeneCountyWebMap/MapServer/2 | NA | N |
| PA | Indiana | NA | Manual | NA | Y |
| PA | Jefferson | NA | Manual | NA | Y |
| PA | McKean | OA | https://github.com/aaronpdennis/pa-county-addresses/raw/master/data/MCKEAN/MCKEAN_addresses.csv | 12/1/2009 | N |
| PA | Mercer | OA | https://github.com/aaronpdennis/pa-county-addresses/raw/master/data/MERCER/MERCER_addresses.csv | 12/1/2009 | N |
| PA | Potter | OA | https://github.com/aaronpdennis/pa-county-addresses/raw/master/data/POTTER/POTTER_addresses.csv | 12/1/2009 | N |
| PA | Tioga | OA | https://github.com/aaronpdennis/pa-county-addresses/raw/master/data/TIOGA/TIOGA_addresses.csv | 12/1/2009 | N |
| PA | Venango | OA | https://github.com/aaronpdennis/pa-county-addresses/raw/master/data/VENANGO/VENANGO_addresses.csv | 12/1/2009 | N |
| PA | Warren | NA | Manual | NA | Y |
| PA | Washington | NA | Manual | NA | Y |
| PA | Westmoreland | NA | Manual | NA | Y |
| WV | Doddridge | OA | ftp://ftp.wvgis.wvu.edu/pub/Clearinghouse/location/AddressSites/County/SAMSii_Doddridge_WVGISTC_042519_utm83_shp.zip | 4/26/2018 | N |
| WV | Gilmer | OA | ftp://ftp.wvgis.wvu.edu/pub/Clearinghouse/location/AddressSites/County/SAMSii_Gilmer_WVGISTC_042519_utm83_shp.zip | 4/26/2018 | N |
| WV | Harrison | OA | ftp://ftp.wvgis.wvu.edu/pub/Clearinghouse/location/AddressSites/County/SAMSii_Harrison_WVGISTC_042519_utm83_shp.zip | 4/26/2018 | N |
| WV | Jackson | OA | ftp://ftp.wvgis.wvu.edu/pub/Clearinghouse/location/AddressSites/County/SAMSii_Jackson_WVGISTC_042519_utm83_shp.zip | 4/26/2018 | N |
| WV | Kanawha | OA | ftp://ftp.wvgis.wvu.edu/pub/Clearinghouse/location/AddressSites/County/SAMSii_Kanawha_WVGISTC_042519_utm83_shp.zip | 4/26/2018 | N |
| WV | Lewis | OA | ftp://ftp.wvgis.wvu.edu/pub/Clearinghouse/location/AddressSites/County/SAMSii_Lewis_WVGISTC_042519_utm83_shp.zip | 4/26/2018 | N |
| WV | Marion | OA | ftp://ftp.wvgis.wvu.edu/pub/Clearinghouse/location/AddressSites/County/SAMSii_Marion_WVGISTC_042519_utm83_shp.zip | 4/26/2018 | N |
| WV | Marshall | OA | ftp://ftp.wvgis.wvu.edu/pub/Clearinghouse/location/AddressSites/County/SAMSii_Marshall_WVGISTC_042519_utm83_shp.zip | 4/26/2018 | N |
| WV | Pocahontas | OA | ftp://ftp.wvgis.wvu.edu/pub/Clearinghouse/location/AddressSites/County/SAMSii_Pocahontas_WVGISTC_042519_utm83_shp.zip | 4/26/2018 | N |
| WV | Preston | OA | ftp://ftp.wvgis.wvu.edu/pub/Clearinghouse/location/AddressSites/County/SAMSii_Preston_WVGISTC_042519_utm83_shp.zip | 4/26/2018 | N |
| WV | Putnam | OA | ftp://ftp.wvgis.wvu.edu/pub/Clearinghouse/location/AddressSites/County/SAMSii_Putnam_WVGISTC_042519_utm83_shp.zip | 4/26/2018 | N |
| WV | Raleigh | OA | ftp://ftp.wvgis.wvu.edu/pub/Clearinghouse/location/AddressSites/County/SAMSii_Raleigh_WVGISTC_042519_utm83_shp.zip | 4/26/2018 | N |
| WV | Randolph | OA | ftp://ftp.wvgis.wvu.edu/pub/Clearinghouse/location/AddressSites/County/SAMSii_Randolph_WVGISTC_042519_utm83_shp.zip | 4/26/2018 | N |
| WV | Ritchie | OA | ftp://ftp.wvgis.wvu.edu/pub/Clearinghouse/location/AddressSites/County/SAMSii_Ritchie_WVGISTC_042519_utm83_shp.zip | 4/26/2018 | N |
| WV | Taylor | OA | ftp://ftp.wvgis.wvu.edu/pub/Clearinghouse/location/AddressSites/County/SAMSii_Taylor_WVGISTC_042519_utm83_shp.zip | 4/26/2018 | N |
| WV | Tyler | OA | ftp://ftp.wvgis.wvu.edu/pub/Clearinghouse/location/AddressSites/County/SAMSii_Tyler_WVGISTC_042519_utm83_shp.zip | 4/26/2018 | N |
| WV | Wetzel | OA | ftp://ftp.wvgis.wvu.edu/pub/Clearinghouse/location/AddressSites/County/SAMSii_Wetzel_WVGISTC_042519_utm93_shp.zip | 4/26/2018 | N |
| WV | Wirt | OA | ftp://ftp.wvgis.wvu.edu/pub/Clearinghouse/location/AddressSites/County/SAMSii_Wirt_WVGISTC_042519_utm83_shp.zip | 4/26/2018 | N |
| WV | Wood | OA | ftp://ftp.wvgis.wvu.edu/pub/Clearinghouse/location/AddressSites/County/SAMSii_Wood_WVGISTC_042519_utm83_shp.zip | 4/26/2018 | N |
